# Different virulence of porcine and porcine-like bovine rotavirus strains with genetically nearly identical genomes in piglets and calves

**DOI:** 10.1186/1297-9716-44-88

**Published:** 2013-10-01

**Authors:** Jun-Gyu Park, Hyun-Jeong Kim, Jelle Matthijnssens, Mia Madel Alfajaro, Deok-Song Kim, Kyu-Yeol Son, Hyoung-Jun Kwon, Myra Hosmillo, Eun-Hye Ryu, Ji-Yun Kim, Rohani B Cena, Ju-Hwan Lee, Mun-Il Kang, Sang-Ik Park, Kyoung-Oh Cho

**Affiliations:** 1 Laboratory of Veterinary Pathology, College of Veterinary Medicine, Chonnam National University, Gwangju 500-757, Republic of Korea; 2 Laboratory of Clinical and Epidemiological Virology, Department of Microbiology and Immunology, Rega Institute for Medical Research, University of Leuven, Leuven, Belgium; 3 Bioindustry Research Center, Korea Research Institute of Bioscience and Biotechnology, Jeongeup 580-185, Republic of Korea; 4 Chonnam National University Veterinary Teaching Hospital, Gwangju 500-757, Republic of Korea

## Abstract

Direct interspecies transmissions of group A rotaviruses (RVA) have been reported under natural conditions. However, the pathogenicity of RVA has never been directly compared in homologous and heterologous hosts. The bovine RVA/Cow-tc/KOR/K5/2004/G5P[7] strain, which was shown to possess a typical porcine-like genotype constellation similar to that of the G5P[7] prototype RVA/Pig-tc/USA/OSU/1977/G5P9[7] strain, was examined for its pathogenicity and compared with the porcine G5P[7] RVA/Pig-tc/KOR/K71/2006/G5P[7] strain possessing the same genotype constellation. The bovine K5 strain induced diarrhea and histopathological changes in the small intestine of piglets and calves, whereas the porcine K71 strain caused diarrhea and histopathological changes in the small intestine of piglets, but not in calves. Furthermore, the bovine K5 strain showed extra-intestinal tropisms in both piglets and calves, whereas the porcine K71 strain had extra-intestinal tropisms in piglets, but not in calves. Therefore, we performed comparative genomic analysis of the K71 and K5 RVA strains to determine whether specific mutations could be associated with these distinct clinical and pathological phenotypes. Full-length sequencing analyses for the 11 genomic segments for K71 and K5 revealed that these strains were genetically nearly identical to each other. Two nucleotide mutations were found in the 5′ untranslated region (UTR) of NSP5 and the 3′ UTR of NSP3, and eight amino acid mutations in VP1-VP4 and NSP2. Some of these mutations may be critical molecular determinants for RVA virulence and/or pathogenicity.

## Introduction

Group A rotavirus (RVA), a member of the *Reoviridae* family, is one of the major pathogens that causes severe and acute dehydrating diarrhea in young children and in a wide variety of domestic animals [1-3]. The rotavirus genome is enclosed in three concentric layers and is comprised of 11 segments of double-stranded (ds) RNA, encoding six structural proteins (VP1-4, VP6 and VP7) and five or six nonstructural proteins (NSP1-NSP5/6) [1,2,4]. This unique segmented nature of the RVA genomes allows for the occurrence of reassortment between different viruses during co-infection in a single cell and induction of progeny viruses with novel or atypical phenotypes [1,2,4].

A whole genome-based genotyping classification system for RVA has been proposed by the Rotavirus Classification Working Group (RCWG), which is based on nucleotide percentage identity cut-off values for each of the 11 RVA genomic segments [5-7]. Among these genotypes, the G (for glycoprotein) and P (for protease-sensitive) genotypes for the VP7 and VP4 outer capsid proteins, respectively, are the most important and frequently analyzed for rotavirus classifications, as they possess important immunogenic epitopes that are relevant for immune protection and vaccine development [1,8]. In extensive genomic studies, 27 G and 37 P genotypes have been identified globally, with various combinations of G and P genotypes distributed across humans and animals [7,9]. The most common G serotypes of RVA found in pigs are G3, G4, G5, G9 and G11 in combination with two dominant P genotypes, P[6] and P[7] [10-12]. In calves, the G6, G8, and G10 genotypes are the major types in combination with either the P[1], P[5] or P[11] genotype [13,14].

There is increasing evidence that the transmission of RVA can occur from animal to human as well as from animal to animal by the contribution of one or several genes to reassortants, which is often combined with a reshuffling of genes into several reassortants [15-21]. Although it is still unclear what determines RVA host range restriction, several studies using different RVA strains and different animal models have implicated at least six rotaviral genome segments in host range restriction and virulence (VP4, VP7, VP3, NSP1, NSP2 and NSP4) [22-28]. Furthermore, there are several reports of direct transmission of RVA strains containing all 11 genome segments from heterologous species in nature. Complete genome analyses of equine RVA strain RVA/Horse-tc/GBR/H-1/1975/G5P[7] revealed a likely porcine-to-equine interspecies transmission [29]. The human RVA strain RVA/Human-wt/BEL/B4106/2000/G3P[14], detected and isolated from a child with enteritis, was shown to possess the same genotype constellation as a lapine G3P[14] RVA strain RVA/Rabbit-tc/ITA/30-96/1996/G3P[14] [30]. Whole genome sequence and phylogenetic analyses have revealed that the human G3P[3] RVA strains RVA/Human-tc/ISR/Ro1845/1985/G3P[3] and RVA/Human-tc/USA/HCR3A/1984/G3P[3] are closely related to the canine RVA/Dog-tc/USA/CU-1/1982/G3P[3], RVA/Dog-tc/AUS/K9/1981/G3P[3] and RVA/Dog-tc/USA/A79-10/1979/G3P[3] RVA strains, and the feline RVA/Cat-tc/AUS/Cat97/1984/G3P[3] RVA strain in all the genome segments [31]. These reports further suggested that direct transmission of heterologous RVA strains can naturally induce gastroenteritis in a heterologous host [30,32]. However, little is known about the exact pathology of these strains in both homologous and heterologous hosts under natural and experimental conditions.

Generally, RVA have been thought to cause only gastrointestinal tract infection, especially in the small intestine. However, increasing evidence indicates that RVA can cause viremia and extra-intestinal infections in humans [33,34]. These data suggest that RVA can escape from the intestinal lesions and routinely spread to extra-intestinal organs [33,34]. In experimental animal models, it has been clearly demonstrated that RVA cause not only gastrointestinal infections but also extra-intestinal infections [34-37], which are caused by viremia [37-40]. These experiments were performed with homologous [36], heterologous [35-38] or reassortant RVA strains [39,40]. However, there is a paucity of direct comparison data on virulence and intestinal and extra-intestinal pathogenicity of RVA strains in their own homologous and heterologous hosts.

Porcine and bovine RVA are important pathogens because of their significant economic impact on the livestock industry as well as their crucial role as reservoirs for human infections [5,11,41]. Therefore, we previously investigated the causes of porcine and bovine diarrhea illnesses and characterized multiple RVA isolates from diarrheic fecal samples [11,19,20,32]. Among the Korean RVA isolated, the bovine RVA strains RVA/Cow-tc/KOR/K5/2004/G5P[7] and RVA/Cow-tc/KOR/K8/2005/G5P[7] isolated from diarrheic calves have a remarkably similar genotype constellation as the porcine G5P[7] RVA strain OSU [32]. This prompted us to investigate whether this heterologous strain K5 could efficiently infect and induce diarrhea and pathology in the intestinal and extra-intestinal tracks of its homologous (piglets) and heterologous (calves) host. To assess the cross-species pathogenicity of the bovine K5 RVA strain in calves and piglets, the porcine G5P[7] OSU-like RVA strain RVA/Pig-tc/KOR/K71/2006/G5P[7] isolated from a diarrheic piglet was also used for comparison in this study. From these experimentally infected animals, various tissues and organs, fecal and nasal swabs and blood were sampled and analyzed for morphological changes, antigen distribution using an immunofluorescence assay (IFA) and viral RNA presence by RT-PCR and real-time RT-PCR. To better understand the potential consequences of viral genetic variations on infection characteristics, the genomic polymorphisms between the porcine K71 and bovine K5 strains were compared. The findings from our study provide insight into the possible functional contributions of viral genomic polymorphisms in the virulence and pathogenicity of heterologous RVA strains.

## Materials and methods

### Origin of virus strains

The bovine K5 and porcine K71 RVA strains were isolated from diarrheic fecal samples of a piglet and a calf, respectively [11,32]. Both strains were passaged eight times in a monolayer of TF-104 cells (a cloned derivative of MA-104 monkey kidney cells) including the initial adaptation and triple plaque purification prior to characterization. Virus titers were assessed by cell cultured immunofluorescence (CCIF) assay using a monoclonal antibody against the VP6 protein of the OSU porcine strain, and were expressed as fluorescence focus units per milliliter (FFU/mL).

### RNA extraction, reverse transcription-PCR (RT-PCR), and DNA sequencing

For each strain, 200 μL of cell culture supernatant was used to extract viral RNA using the Accuprep^®^ Viral RNA Extraction kit (Bioneer, Daejon, South Korea) according to the manufacturer’s instructions. The extracted RNA was denatured at 94 °C for 10 min and one-step RT-PCR was performed as described elsewhere [39,40,42]. The sequencing primer pairs are shown in the Additional file 1[5,20,30,43-46]. Briefly, 5 μL of denatured RNA was added to a 45 μL RT-PCR mixture containing 5 μL of 10 X PCR buffer [100 mM Tris–HCl (pH 8.3), 500 mM KCl, 15 mM MgCl_2_, 0.01% gelatin], 5 μL of MgCl_2_ (25 mM), 1 μL of 10 mM dNTPs, 1 μL of the upstream primer (50 pmol), 1 μL of the downstream primer (50 pmol), 0.5 μL of ImProm-II™ Reverse Transcriptase (5.0 U) (Promega, Madison, WI, USA), 0.5 μL of RNasin-RNase inhibitor (10 U) (Promega), 0.5 μL of *Taq* polymerase (2.5 U) (Promega) and 30.5 μL water. The mixture was incubated for 60 min at 42 °C, preheated for 5 min at 94 °C, subjected to 35 cycles of 1 min at 94 °C, 1 min at 45 ~ 55 °C depending on the primer sets and 2 min at 72 °C, and a final extension of 7 min at 72 °C.

RT-PCR products amplified by primer pairs specific to each genomic segment were purified using a QIAEX II Gel Extraction kit (Qiagen, Valencia, CA, USA) according to the manufacturer’s instructions. The extracted PCR products were ligated into the pGEM-T Easy Vector Systems (Promega), and were sub-cloned into home-made XL1-Blue competent cells. Individual colonies were grown and plasmid was purified using Hybrid-Q™ Plasmid (GeneAll, Seoul, South Korea). DNA sequencing was carried out using an ABI System 3700 automated DNA sequencer (Applied Biosystems, Foster City, CA, USA).

### Determination of the 3′ and 5′ terminal sequences of K71 and K5 rotavirus strains

#### Poly (A) tailing reaction and 3′ and 5′ cDNA synthesis

Total RNA was extracted from a starting volume of 200 μL cell lysates by AccuPrep^®^ Viral RNA extraction kit (Bioneer) according to the manufacturer’s instructions. Poly (A) tailing reaction was done using *Escherichia coli* poly (A) polymerase (Ambion, Austin, TX, USA) according to the manufacturer’s instructions. cDNA synthesis was performed using the SMARTer™ Rapid Amplification of cDNA Ends (RACE) cDNA amplification kit (Clontech, Mountain View, CA, USA). For the one reaction generation of 3′ RACE-ready cDNA, 3.75 μL of the poly A tailed RNA and 1.0 μL of 3′-cDNA Synthesis (CDS) Primer A were mixed and incubated at 72 °C for 3 min, followed by cooling the tubes at 42 °C for 2 min using a thermo cycler. The denatured RNA was mixed with a reaction mixture composed of 2.0 μL 5 X First-Strand Buffer, 1.0 μL dithiothreitol (DTT) (20 mM), 1.0 μL dNTP mix (10 mM), 0.25 μL RNase inhibitor (40 U/μL), and 1.0 μL SMARTScribe™ Reverse Transcriptase (100 U). Samples were incubated at 42 °C for 90 min and were heated to 70 °C for 10 min using a thermo cycler. The synthesized cDNA was diluted with 7 μL of Tricine-EDTA buffer and was used immediately or stored in -20 °C prior to RACE PCR.

For a more efficient amplification of 5′ RACE cDNA for both strains, cDNA synthesis was carried out using random primers. In brief, 2.75 μL of total RNA was mixed with 1.0 μL of 10X random primer, incubated at 72 °C for 3 min, and cooled at 22 °C for 2 min. The preheated RNA was mixed with a reaction mixture consisting of 2 μL 5 X First-Strand Buffer, 1 μL DTT (20 mM), 1 μL dNTP mix (10 mM), 0.25 μL RNase inhibitor, and 1.0 μL SMARTer™ II A Oligonucleotide (12 uM). The reaction was incubated at room temperature (RT) for 10 min before adding 1.0 μL of SMARTer™ reverse transcriptase (100 U/μL). The reaction was incubated at 42 °C for 90 min and was then heated to 70 °C for 10 min. The cDNA was diluted with 7 μL of Tricine-EDTA buffer and was then used immediately or stored at -20 °C prior to RACE PCR.

#### RACE PCR, cloning and sequencing

Generation of 3′ and 5′ RACE PCR reactions were carried out using Advantage 2 Polymerase Mix (Clontech); 5.0 μL of 3′/5′-RACE-Ready cDNA, 32.0 μL of PCR-grade water, 5.0 μL of 10 X Advantage 2 PCR Buffer, 1.0 μL dNTP Mix (10 mM), 5.0 μL Universal Primer Mix (10 X), 1.0 μL of 50 pmol gene-specific primer (GSP) for 3′ and 5′ RACE (see Additional file 1), and 1.0 μL of 50 X Advantage 2 Polymerase Mix. The mixture was commenced in the following thermal cycling program: 5 cycles of 94 °C for 30 s and 72 °C for 3 min; 5 three-step cycles of 94 °C for 30 s, 65 °C (applied melting temperature of GSP) for 30 s, and 72 °C for 3 min; 25 three-step cycles of 94 °C for 30 s, 60 °C (applied lowered Tm values of GSP by 3 °C to 5 °C) for 30 s, and 72 °C for 3 min.

The RACE PCR products were separated on a 1.2% (m/v) agarose gel, and the bands were excised and purified using the Purelink™ Quick Gel Extraction kit (Invitrogen, Lohne, Germany). The RACE PCR products were ligated into a pGEM-T Easy Vector Systems (Promega) and were sub-cloned into home-made XL1-Blue competent cells. Individual colonies were grown and plasmid was purified using Hybrid-Q™ Plasmid (GeneAll). Sequencing was performed using an ABI System 3700 automated DNA sequencer (Applied Biosystems).

### Molecular characterization

To generate complete nucleotide sequences for each genomic segment, both 5′ and 3′ end sequences of each genomic segment were assembled with the nucleotide sequence of the internal region sequenced with GSP. The full-length nucleotide sequences of each genomic segment of both porcine K71 and bovine K5 strains were compared with those of the other known RVA strains using the DNAsis MAX DNA Basic module (MiraiBio, Alameda, CA, USA). Phylogenetic and molecular evolutionary analyses based on the nucleotide alignments were constructed using the neighbor-joining method [47]. Genetic distances were calculated using the Kimura-2 correction parameter at the nucleotide level, and the phylogenetic trees were constructed using the neighbor-joining method with 1000 bootstrap replicates. A sequence similarity search for the porcine K71 and bovine K5 strains with those of the other known RVA strains (see Additional file 2) was performed using the homology and distance matrices method of DNAMAN version 6.0 program (Lynnon, Vaudreuil, PQ, Canada).

### Genotype assignment

The genotypes for the 11 genome segments of the porcine K71 and bovine K5 rotavirus strains were determined according to the genotyping recommendations of the Rotavirus Classification Working Group (RCWG) [6].

### Genebank accession numbers

The Genbank accession numbers for the 11 genome segments of strains used in this study are shown in Additional file 2.

### Animals and experiment design

To evaluate the pathogenicity of the bovine K5 and porcine K71 strains in piglets, 20 3-day-old piglets obtained from sows by hysterectomy were used. Eight piglets each were orally inoculated with 4 mL of porcine K71 (1.1 × 10^7^ FFU/mL) or bovine K5 (2.5 × 10^7^ FFU/mL) strains, respectively (Tables 1 and 2). As negative controls, piglets were inoculated with 4 mL of the mock-infected TF-104 cell culture supernatant, or chloroform-inactivated porcine K71 or bovine K5 strains, respectively (Tables 1 and 2). All piglets were fed with sterilized commercialized milk (Sprayfo^®^, Sloten B.V., Antwerpenweg, Netherlands).

**Table 1:** Summary of incidence of diarrhea, fecal virus shedding, and viremia in the colostrum-deprived piglets inoculated with a porcine G5P[7] K71 strain.

**Piglet no.**	**dpi at euthanasia**	**Diarrhea onset dpi (duration)**	**RT-PCR onset dpi (duration)**		**Nested-PCR onset dpi (duration)**	
			**Feces**	**Serum**	**Feces**	**Serum**
1	1	1 (1)	1 (1)	None	1 (1)	None
2	1	1 (1)	1 (1)	None	1 (1)	None
3	3	1 (3)	1 (2)	2 (1)	1 (3)	2 (2)
4	3	1 (3)	1 (2)	2 (1)	1 (3)	2 (2)
5	5	1 (5)	1 (3)	2 (1)	1 (5)	2 (2)
6	5	1 (5)	1 (4)	2 (2)	1 (5)	2 (3)
7	7	1 (7)	1 (5)	2 (3)	1 (7)	2 (3)
8	14	1 (14)	1 (7)	2 (3)	1 (14)	2 (3)
9^a^	2	None	None	None	None	None
10^b^	2	None	None	None	None	None

^a^Mock-infected TF-104 cell culture supernatant.

^b^Inoculation with a chloroform-inactivated porcine K71 strain.

**Table 2:** Summary of incidence of diarrhea, fecal virus shedding, and viremia in the colostrum-deprived piglets inoculated with a bovine G5P[7] K5 strain.

**Piglet no.**	**dpi at euthanasia**	**Diarrhea onset dpi (duration)**	**RT-PCR onset dpi (duration)**		**Nested-PCR onset dpi (duration)**	
			**Feces**	**Serum**	**Feces**	**Serum**
1	1	1 (1)	1 (1)	None	1 (1)	None
2	1	1 (1)	1 (1)	None	1 (1)	1 (1)
3	3	1 (3)	1 (3)	2 (1)	1 (3)	2 (2)
4	3	1 (3)	1 (2)	2 (2)	1 (3)	2 (2)
5	5	1 (5)	1 (5)	2 (1)	1 (5)	2 (3)
6	5	1 (5)	1 (4)	2 (2)	1 (5)	2 (3)
7	7	1 (7)	1 (6)	2 (4)	1 (7)	2 (4)
8	14	1 (14)	1 (10)	2 (3)	1 (14)	2 (4)
9^a^	2	None	None	None	None	None
10^b^	2	None	None	None	None	None

^a^Mock-infected TF-104 cell culture supernatant.

^b^Inoculation with a chloroform-inactivated bovine K5 strain.

A total of sixteen 2-day-old colostrum-deprived (Cols-D) Holstein calves were used to evaluate the pathogenicity of the porcine K71 and bovine K5 strains. Five and seven calves were orally inoculated with 40 mL supernatants of porcine K71 (1.1 × 10^7^ FFU/mL) and bovine K5 (2.5 × 10^7^ FFU/mL) strains, respectively (Tables 3 and 4). The calves serving as negative controls were inoculated with 40 mL of the mock-infected TF-104 cell culture supernatant, or chloroform-inactivated porcine K71 or bovine K5 strains, respectively (Tables 3 and 4). All calves were fed with sterilized commercialized milk (Sprayfo^®^, Sloten B.V., Antwerpenweg, Netherlands).

**Table 3:** Summary of incidence of diarrhea, fecal virus shedding and viremia in the colostrum-deprived calves inoculated with a porcine G5P[7] K71 strain.

**Calf no.**	**dpi at euthanasia**	**Diarrhea onset dpi (duration)**	**RT-PCR onset dpi (duration)**		**Nested-PCR onset dpi (duration)**	
			**Feces**	**Serum**	**Feces**	**Serum**
1	1	None	1 (1)	None	1 (2)	None
2	3	None	1 (1)	None	1 (2)	None
3	5	None	1 (1)	None	1 (1)	None
4	7	None	1 (1)	None	1 (2)	None
5	14	None	1 (1)	None	1 (1)	None
6^a^	2	None	None	None	None	None
7^b^	3	None	None	None	None	None

^a^Mock-infected TF-104 cell culture supernatant.

^b^Inoculation with a chloroform-inactivated porcine K71 strain.

**Table 4:** Summary of incidence of diarrhea, fecal virus shedding, and viremia in the colostrum-deprived calves inoculated with a bovine G5P[7] K5 strain.

**Calf no.**	**dpi at euthanasia**	**Diarrhea onset dpi (duration)**	**RT-PCR onset dpi (duration)**		**Nested-PCR onset dpi (duration)**	
			**Feces**	**Serum**	**Feces**	**Serum**
1	1	1 (1)	1 (1)	1 (1)	1 (1)	1 (1)
2	3	1 (3)	1 (3)	2 (2)	1 (3)	2 (2)
3	5	1 (5)	1 (5)	1 (4)	1 (5)	1 (4)
4	6	1 (6)	1 (6)	1 (5)	1 (6)	1 (6)
5	7	1 (7)	1 (7)	2 (5)	1 (7)	2 (5)
6	12	1 (12)	1 (10)	1 (4)	1 (14)	1 (5)
7	14	1 (14)	1 (9)	2 (3)	1 (12)	2 (4)
8^a^	2	None	None	None	None	None
9^b^	3	None	None	None	None	None

^a^Mock-infected TF-104 cell culture supernatant.

^b^Inoculation with a chloroform-inactivated bovine K5 strain.

After inoculation, color and consistency of feces obtained from each calf and piglet were evaluated daily. The consistency of the feces was scored on a scale of 0–4, with 0 representing firm; 1, pasty; 2, semi-mucoid; 3, liquid and 4, profuse diarrhea [39,40,42,48]. Fecal and blood samples were collected daily from each calf and piglet before and after inoculation, as described previously [39,40,42,48]. The inoculated calves and piglets were euthanized at given times (Tables 1, 2, 3 and 4). Calves and piglets inoculated with the mock- or inactivated virus were euthanized at 2 or 3 day-post inoculation (dpi).

Necropsy was immediately performed after euthanasia. During necropsy, the intestinal tract, mesenteric lymph node (MLN), nasal turbinate, trachea, pancreas, lung, liver, spleen, brain, kidney, heart, urinary bladder, and choroid plexus were collected from experimental calves and piglets [39,40]. All organs were immediately placed in 10% buffered formalin for histological examination. Blood samples were collected from the jugular vein of the calves and via the intra-cardiac route for the piglets.

To evaluate the RVA-antigen distribution, intestinal and extra-intestinal organs from virus-inoculated and mock-inoculated experimental animals were sampled, embedded in Optimum Cutting Temperature compound, immediately snap-frozen in liquid nitrogen and stored at -80 °C [39,40]. Cerebrospinal fluid (CSF) was collected after euthanasia from each experimental animal and stored at -80 °C [39,40]. All samples collected for RT-PCR and real-time RT-PCR were immediately snap-frozen in liquid nitrogen, and kept at -80 °C until use [39,40]. All procedures were approved by the Animal Care Committee of Chonnam National University (CNU IACUC-YB-2009-15).

### Examination of small intestinal histopathological changes

The formalin-fixed samples were embedded in paraffin and sectioned. Serial 4 μm sections were stained with Mayer’s hematoxylin and eosin, and were examined microscopically. Histological evaluation was performed in a blind fashion on coded samples and a comparison was made with the sections from the mock-inoculated controls [39,40]. Histopathological lesions of small intestinal villi were scored according to the average villi/crypt (V/C) ratio plus the grade of epithelial cell desquamation, which was measured as follows: V/C ratio, 0 = normal; (V/C ≥ 6:1), 1 = mild; (V/C = 5.0-5.9:1), 2 = moderate; (V/C = 4.0-4.9:1), 3 = marked; (V/C = 3.0-3.9:1), 4 = severe; (V/C ≤ 3.0:1), and desquamation grade, 0 = normal (no desquamation), 1 = mild (a few desquamated cells of tip villous epithelium), 2 = moderate (desquamation of upper villous epithelium), 3 = marked (desquamation of lower villous epithelium), and 4 = severe (desquamation of crypt epithelium). These mean lesion changes were determined by measuring 10 randomly selected villi and crypts on intestinal sections, respectively, similar to the methods described previously [39,40,42,48].

### Examination of rotavirus antigen by IFA

For assessment of the antigen distribution of rotavirus, IFA was performed in each organ sampled from virus-inoculated and mock-inoculated animals, as described elsewhere [35,39,40,42,48]. Briefly, cut frozen sections from experimental animals were fixed in 100% cold acetone for 10 min and were allowed to completely air-dry. Slides were washed twice with phosphate buffered saline (PBS, pH 7.2), and incubated for 2 h at RT with a 1:100 dilution of monoclonal anti-VP6 antibody diluted in PBS (pH 7.2). Slides were washed twice with PBS (pH 7.2), and incubated with goat anti-mouse Ig conjugated to fluorescein isothiocyanate (Jackson ImmunoResearch Labs, Baltimore, MD, USA) diluted 1:100 in PBS (pH 7.2) for 1 h at RT. Following incubation, the slides were washed twice with PBS (pH 7.2). Slides were incubated with propidium iodide diluted in 500 nM PBS (pH 7.2) for 10 min at RT as a nucleic acid stain. Slides were washed twice with PBS (pH 8.0), and covered with 60% glycerin in PBS (pH 8.0), and glass cover slips. Fluorescence of the samples was examined microscopically under ultraviolet illumination (Leica Microsystems, Wetzlar, Germany). To calculate the number of antigen-positive cells in the organs or tissues, 10 fields per section were analyzed, using a 40× objective and a 10× eyepiece, yielding a final magnification of 400×. Total counts of antigen-positive cells were calculated as mean values.

### Examination of viral RNA in the fecal and serum samples by RT-PCR and nested-PCR

For the detection of viral RNA in the fecal and serum specimens from each experimental animal, RT-PCR and nested-PCR with primer pairs specific to the RVA VP6 gene were performed as previously described [39,40]. As negative controls, fecal and serum specimens sampled from mock-inoculated animals were used. The PCR products were visualized on 1.2% agarose gels stained with ethidium bromide.

### Quantificaiton of viral RNA by real time RT-PCR using SYBR green chemistry

The one-step real-time RT-PCR assay was performed with a primer pair specific to the RVA VP6 gene, as described previously [39,40,49]. Briefly, all tissue and fluid samples from the experimental animals were individually weighed, homogenized or vortexed at a 1:10 dilution in 0.01 M PBS and were centrifuged (tissues 13 000 × *g* for 3 min; fecal samples 5000 × *g* for 10 min). The supernatants along with the remaining bulk samples were collected and stored at -80 °C prior to analysis. After extracting total RNA from supernatants, each real time RT-PCR reaction was performed using a Rotor-Gene Real-Time Amplification system (Corbett Research, Mortlake, Australia) and SensiMix one-step RT-PCR kit with SYBR Green (Quantace, London, UK) in a final volume of 25 μL containing 5 μL of the RNA template, 12.5 μL SensiMix one-step mixture, 1 μL each of 0.5 M forward and reverse primers (final concentration of each primer: 20 nM), 0.5 μL of 50 X SYBR Green solution (final concentration: 1 X), 0.5 μL of RNase inhibitor (final concentration: 10 U), 0.5 μL of MgCl_2_ (final concentration: 4.0 mM) and 4 μL of RNase-free water [39,40,49]. Reverse transcription was carried out at 50 °C for 30 min, followed by the activation of the hot-start DNA polymerase at 95 °C for 15 min and 40 three-step cycles: 95 °C for 15 s, 51 °C for 30 s, and 72 °C for 1 min. Quantification of virus RNA copies was carried out using a standard curve derived from serial 10-fold dilutions of the in vitro transcription of complementary RNA (cRNA) amplified in separate PCR tubes. Rotorgene 6000^®^ (Corbett Research) software was used for the calculation of the amount of rotavirus-specific RNA in the samples. The threshold was defined automatically in the initial exponential phase, reflecting the highest amplification rate. With regards to the crossing points resulting from the amplification curves and this threshold, a direct relation between the cycle number and the log concentration of RNA molecules initially present in the RT-PCR reaction was evident. By linear regression analysis of these data, Rotorgene 6000^®^ software set up a standard curve, which enabled the determination of the concentration of RNA present in the samples.

## Results

### Sequence and phylogenetic analyses

Three full-length (VP7, NSP2 and NSP5) and eight partial-length genomic sequences (VP1-VP4, VP6, NSP1, NSP3 and NSP4) from the bovine RVA strain K5 were characterized previously [32]. For the porcine K71 strain, the full-length VP7 and partial VP4 genomic sequences were also determined [11]. In this study, the full-length genomic sequences of both strains were sequenced by RT-PCR assays with GSP specific to both 5′ and 3′ end sequences. In addition, 5′ and 3′ RACE for the 11 genome segments of both strains was performed to obtain the full-length nucleotide sequences of both strains.

The full-length nucleotide sequences of all 11 genomic segments of both porcine K71 and bovine K5 strains were analyzed and compared with those of the reference RVA strains. Generally, both strains shared 99.6-100% nucleotide identities of all 11 genomic segments (Table 5). The gene segments for K71 and K5 had the highest nucleotide identities with porcine RVA strain OSU (VP1-VP4, VP7, NSP1-NSP3), porcine strain JL94 (VP6), porcine strain RMC321 (NSP4), and panda strain CH-1 (NSP5) (Table 5). The genotype constellations of strains K71 and K5 were identical; G5-P[7]-I5-R1-C1-M1-A1-N1-T1-E1-H1, which is typical for porcine G5P[7] RVA strains, such as OSU (see Additional file 3). Phylogenetic analyses also demonstrated that all 11 genomic segments of both porcine K71 and bovine K5 strains clustered with those of porcine and porcine-like RVA strains (see Additional file 4).

**Table 5:** Comparison of full-length nucleotide sequences of all eleven genomic segments between the Korean porcine K71 and the Korean bovine K5 strains, and between the Korean and other known strains.

**Genomic segments**	**% nucleotide identities between**		
	**K5 and K71 strains**	**K5 and other known strain**	**K71 and other known strain**
		**(Strain exhibiting the highest identity)**	**(Strain exhibiting the highest identity)**
VP7	100	99.72 (OSU)	99.72 (OSU)
VP4	99.96	99.49 (OSU)	99.53 (OSU)
VP6	100	99.63 (JL94)	99.63 (JL94)
VP1	99.94	99.88 (OSU)	99.88 (OSU)
VP2	99.93	99.96 (OSU)	99.89 (OSU)
VP3	99.96	99.54 (OSU)	99.62 (OSU)
NSP1	100	99.62 (OSU)	99.62 (OSU)
NSP2	99.62	99.62 (OSU)	99.43 (OSU)
NSP3	99.81	96.84 (OSU)	97.02 (OSU)
NSP4	100	95.47 (RMC321)	95.47 (RMC321)
NSP5	99.85	98.80 (CH-1)	99.95 (CH-1)

### Intestinal pathogenicity in piglets and calves

The above results indicate that the porcine K71 and bovine K5 strains possessed genomic constellations typical to porcine RVA strains such as OSU. Therefore, we determined whether these strains show different intestinal pathogenicity in piglets and calves. The porcine K71 strain induced continuous diarrhea in all inoculated piglets from 1 dpi to the termination of the experiment (Table 1), but did not cause any diarrhea in the inoculated calves (Table 3). Nested PCR assay continuously detected fecal virus shedding from 1 dpi to the termination of the experiment in piglets (Table 1). Although the virus was also detected at 1 dpi in all calves, this viral shedding persisted for only 1 or 2 days (Table 3).

**Figure 1:**
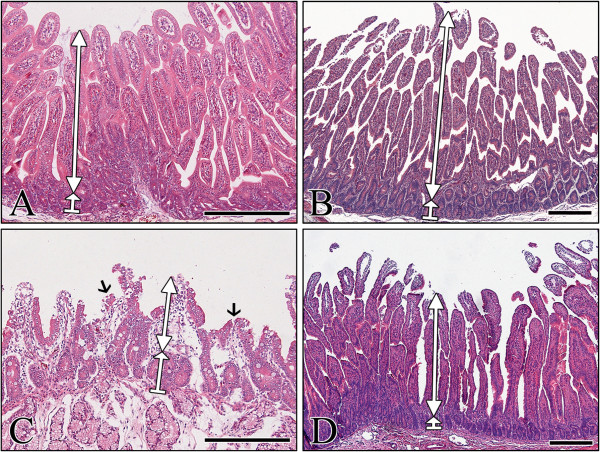
**Histopathological changes in the small intestine of calves and piglets infected with the porcine G5P[7] RVA strain K71. (A** and **B)** Duodenum sampled from a mock-inoculated piglet **(A)** and a calf **(B)** had normal long slender villi (up-down arrow) and short crypt (up-wards arrow) in the mucosal membrane. **(C)** Duodenum sampled from a virus-infected piglet at 3 dpi showed severe villous atrophy (up-down arrow), villous fusions (arrows), and increased crypt depth (up-wards arrow). **(D)** Duodenum sampled from a virus-infected calf at 3 dpi displayed normal long slender villi (up-down arrow) and short crypt (up-wards arrow) in the mucosal membrane. Hematoxylin and eosin stain. Bars denote 400 μm.

**Figure 2:**
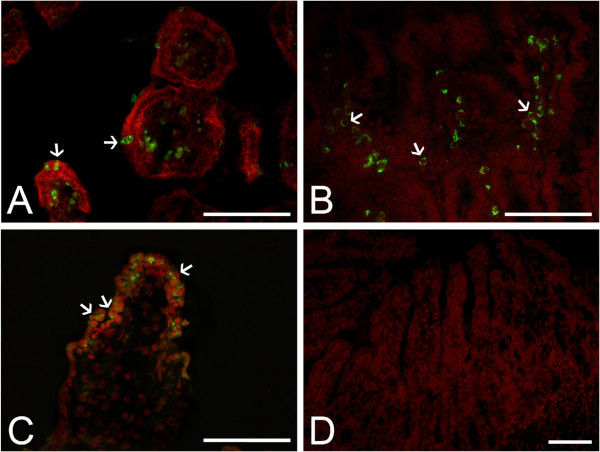
**Distribution of RVA antigen-positive cells of porcine and bovine G5P[7] strains in the duodenum. (A** and **B)** Duodenum sampled at 3 dpi from a piglet **(A)** and a calf **(B)** infected with bovine K5 strain had antigen-positive cells (arrows) in the villi. **(C** and **D)** K71 RVA antigen-positive cells (arrows) were detected in the villi of duodenum sampled from a virus inoculated piglet at 1 dpi **(C)**, but not in those from a virus inoculated calf **(D)**. Indirect immunofluorescence assay with monoclonal antibody against the VP6 protein of strain OSU. Bars denote 100 μm.

Small intestinal lesions typical of RVA infection included desquamation of villi epithelium resulting in villous atrophy and fusion, and crypt hyperplasia. Sequential histopathological lesion changes in the small intestines of piglets and calves infected with each strain are summarized in Additional files 5, 6, 7 and 8. The porcine K71 strain tended to cause a sequential increase of histopathological lesion changes in the small intestine of inoculated piglets, but induced only mild changes in the small intestine of calves (see Additional files 5, 6, 7 and 8 and Figure 1). RVA antigen-positive cell counts in the small intestinal villi were much higher in piglets than in calves (see Additional files 5, 6, 7 and 8 and Figure 2). Mock-inoculated or inactivated-virus-inoculated piglets and calves had no diarrhea, fecal virus shedding, or histopathological changes in the small intestine. From these results, it can be concluded that the porcine K71 strain efficiently infected the small intestines of piglets, but not calves.

**Figure 3:**
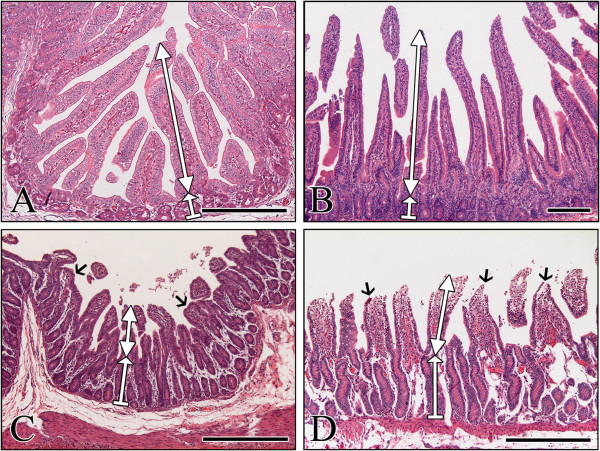
**Histopathological changes in the small intestine of calves and piglets infected with the bovine G5P[7] RVA strain K5. (A** and **B)** Duodenum sampled from mock-inoculated piglet **(A)** and calf **(B)** had normal long slender villi (up-down arrow) and short crypt (up-wards arrow) in the mucosal membrane. **(C)** Duodenum sampled from a virus-infected piglet at 3 dpi showed severe villous atrophy (up-down arrow), villous fusion (arrows), and increased crypt depth (up-wards arrow). **(D)** Duodenum sampled from a virus-infected calf at 3 dpi had severe villous atrophy (up-down arrow), villous fusions (arrows) and increased crypt depth (up-wards arrow). Hematoxylin and eosin stain. Bars denote 400 μm.

In contrast to the porcine K71 strain, the bovine K5 strain induced continuous diarrhea in both virus-inoculated piglets and calves from 1 dpi to the termination of the experiment (Tables 2 and 4). Fecal virus shedding was also continuously detected by nested PCR from K5 strain-inoculated piglets and calves (Tables 2 and 4). The bovine K5 strain tended to cause a sequential increase of small intestinal lesion changes in both virus-inoculated calves and piglets (see Additional files 7 and 8, and Figure 3). RVA antigen-positive cells in the small intestinal villi were detected at high levels at 1 dpi and gradually decreased in both virus-inoculated calves and piglets (see Additional files 7 and 8, and Figure 2). Mock-inoculated or inactivated-virus-inoculated piglets and calves had no diarrhea, fecal virus shedding, or histopathological changes in the small intestine. These results indicate that bovine K5 efficiently infected the small intestines of both calves and piglets.

### Extra-intestinal histopathological lesion changes in piglets and calves

**Figure 4:**
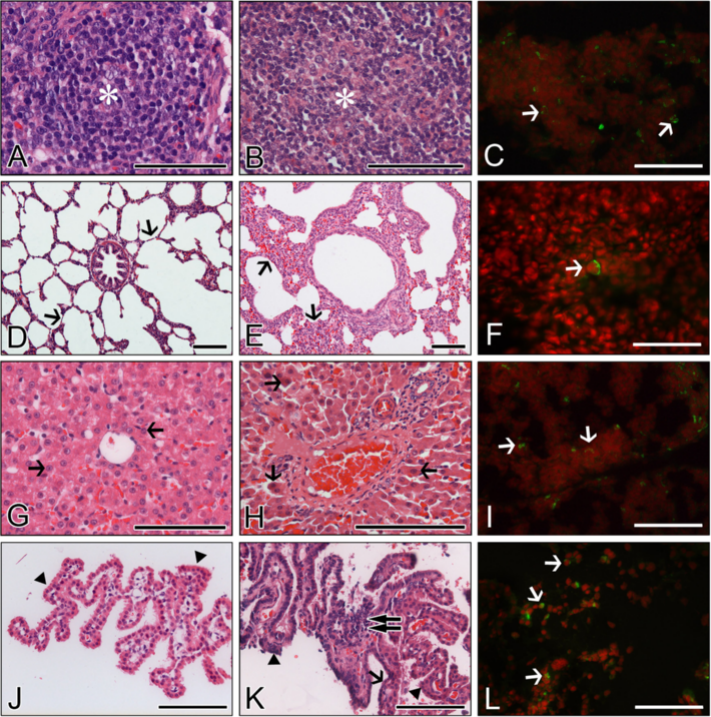
**Extra-intestinal histopathological changes and distribution of antigen-positive cells in piglets infected with porcine G5P[7] RVA strain K71. (A**-**C)** Compared to densely packed lymphocytes (asterisk) in the cortex of normal mesenteric lymph node (MLN) from a mock-inoculated piglet **(A)**, MLN from a virus-inoculated piglet showed lymphoid cell depletion (asterisk) in the cortex **(B)** and RVA antigen-positive cells (arrows) **(C)**. **(D**-**F)** The lung sampled from a mock-inoculated piglet revealed normal thin alveolar wall (arrows) **(D)**, whereas the lung sampled from a virus-inoculated piglet showed interstitial pneumonia (arrows) **(E)** and RVA antigen-positive cells (arrow) **(F)**. **(G**-**I)** Compared to normal fat-storing hepatocytes (arrows) from a mock-inoculated piglet **(G)**, liver sampled from a virus-inoculated piglet showed multiple scattered necrotic hepatocytes (arrows) **(H)** and RVA antigen-positive cells (arrows) **(I)**. **(J**-**L)** Choroid plexus sampled from a mock-inoculated piglet had an intact epithelium (arrowheads) **(J)**, whereas choroid plexus sampled from a virus-inoculated piglet displayed epithelial degeneration (arrowheads) and necrosis (arrows), and lymphoid cell infiltration (double arrow) into the tela choroidea **(H)**, and RVA antigen-positive cells (arrows) **(L)**. Hematoxylin and eosin stain **(A**, **B**, **D**, **E**, **G**, **H**, **J**, and **K)**. Indirect immunofluorescence assay with monoclonal against the VP6 protein of strain OSU **(C**, **F**, **I** and **L)**. Bars denote 100 μm.

**Figure 5:**
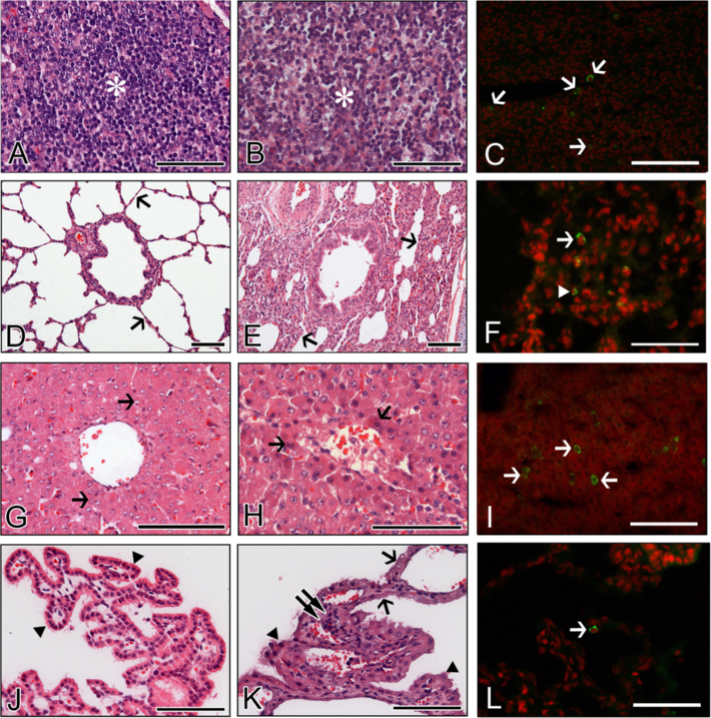
**Extra-intestinal histopathological changes and distribution of antigen-positive cells in piglets infected with bovine G5P[7] RVA strain K5. (A-C)** Compared to densely packed lymphocytes (asterisk) in the cortex of normal mesenteric lymph node (MLN) from a mock-inoculated piglet **(A)**, MLN from a virus-inoculated piglet showed lymphoid cell depletion (asterisk) in the cortex **(B)** and RVA antigen-positive cells (arrows) **(C)**. **(D**-**F)** The lung sampled from a mock-inoculated piglet reveals normal thin alveolar wall (arrows) **(D)**, whereas the lung sampled from a virus-inoculated piglet shows interstitial pneumonia (arrows) **(E)** and an RVA antigen-positive reaction in the pneumocyte (arrowhead) and lymphoid cell (arrow) **(F)**. **(G**-**I)** Compared to normal fat-storing hepatocytes (arrows) from a mock-inoculated piglet **(G)**, liver sampled from a virus-inoculated piglet shows multiple scattered necrotic hepatocytes (arrows) **(H)** and RVA antigen-positive cells (arrows) **(I)**. **(J**-**L)** Choroid plexus sampled from a mock-inoculated piglet had intact epithelium (arrowheads) **(J)**, whereas choroid plexus sampled from a virus-inoculated piglet displayed epithelial degeneration (arrowheads) and necrosis (arrows), and lymphoid cell infiltration (double arrow) into the tela choroidea **(H)**, and RVA antigen-positive cells (arrow) **(L)**. Hematoxylin and eosin stain **(A**, **B**, **D**, **E**, **G**, **H**, **J**, and **K)**. Indirect immunofluorescence assay with monoclonal antibody against the VP6 protein of strain OSU **(C**, **F**, **I**, and **L)**. Bars denote 100 μm.

**Figure 6:**
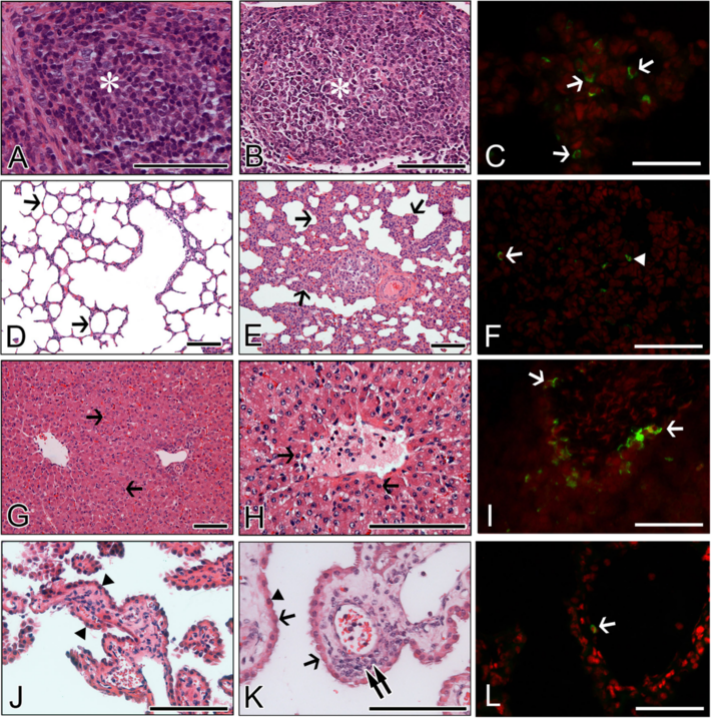
**Extra-intestinal histopathological changes and distribution of antigen-positive cells in calves infected with bovine G5P[7] RVA strain K5. (A**-**C)** Compared to densely packed lymphocytes (asterisk) in the cortex of normal mesenteric lymph node (MLN) from a mock-inoculated calf **(A)**, MLN from a virus-inoculated calf shows lymphoid cell depletion (asterisk) in the cortex **(B)** and RVA antigen-positive cells (arrows) **(C)**. **(D**-**F)** Lung sampled from a mock-inoculated calf reveals normal thin alveolar wall (arrows) **(D)**, whereas lung sampled from a virus-inoculated calf shows interstitial pneumonia (arrows) **(E)** and RVA antigen-positive reaction in the pneumocyte (arrowhead) and lymphoid cell (arrow) **(F)**. **(G**-**I)** Compared to normal hepatocytes (arrows) from a mock-inoculated calf **(G)**, liver sampled from a virus-inoculated calf shows multiple scattered necrotic hepatocytes (arrows) **(H)** and RVA antigen-positive cells (arrows) **(I)**. **(J**-**L)** Choroid plexus sampled from a mock-inoculated calf has intact epithelium (arrowheads) **(J)**, whereas choroid plexus sampled from a virus-inoculated calf displays epithelial degeneration (arrowhead) and necrosis (arrows), and lymphoid cell infiltration (double arrow) into the tela choroidea **(H)**, and RVA antigen-positive cells (arrow) **(L)**. Hematoxylin and eosin stain **(A**, **B**, **D**, **E**, **G**, **H**, **J**, and **K)**. Indirect immunofluorescence assay with monoclonal antibody against the VP6 protein of strain OSU **(C**, **F**, **I**, and **L)**. Bars denote 100 μm.

The above results indicate that the porcine K71 strain efficiently infected the small intestines of piglets, whereas the bovine K5 strain efficiently infected the small intestines of calves and piglets. Furthermore, histological changes in extra-intestinal organs and tissues were examined in piglets and calves inoculated with the porcine K71 or bovine K5 strains. Histopathological changes were absent in the MLN sampled from inactivated virus- or mock-inoculated piglets or calves. The porcine K71 RVA strain induced lymphoid cell depletions with the infiltration of some macrophages and neutrophils in the cortex of MLN obtained from piglets (Figure 4) but not from calves (data not shown). However, the bovine K5 strains induced these histopathological changes in the MLN from both virus-inoculated piglets and calves (Figures 5 and 6). As was seen for the histopathological changes, porcine K71 RVA antigen-positive cells were detected in the MLN of piglets, but not from those of calves (see Additional files 9 and 10, and Figure 4). However, bovine K5 RVA antigen-positive cells were observed in the MLN of both piglets and calves (see Additional files 11 and 12, and Figures 5 and 6).

The porcine K71 strain induced hepatocyte necroses with some macrophages and lymphocyte infiltration in the liver sampled from the virus-infected piglets (Figure 4) but not from virus-infected calves (data not shown). Cells positive for RVA K71 antigen were only detected in the liver from the virus-infected piglets but not in that from the virus-infected calves (see Additional files 9 and 10, and Figure 4). In contrast, the bovine K5 strain induced hepatocyte necroses and antigen-positive cells in the livers from both virus-inoculated piglets and calves (see Additional files 11 and 12, and Figures 5 and 6).

The porcine K71 strain caused lung lesions characterized by mild to moderate thickening of alveolar walls by hyperplasia of type II pneumocytes, and infiltration of macrophages and lymphoid cells into the alveolar interstitium in the virus-inoculated piglets (Figure 4) but not in the calves (data not shown). Cells positive for K71 RVA antigen were only detected in the lungs from the virus-inoculated piglets but not in those from the virus-inoculated calves. The bovine K5 strains elicited these lesions in the lungs of both virus-inoculated piglets and calves (see Additional files 11 and 12, and Figures 5 and 6).

In the choroid plexus, the porcine K71 strain induced limited epithelial degeneration and necroses with mild lymphoid cell infiltration in the tela choroidea of the piglets (Figure 4) but not in that of calves (data not shown). Cells positive for K71 porcine RVA antigen were only detected in the tela choroidea of piglets but not in that of calves (see Additional files 9 and 10, and Figure 4). The bovine K5 strain cited above induced histopathological changes and antigen-positive cells in the tela choroidea of both virus-inoculated piglets and calves (see Additional files 11 and 12, and Figures 5 and 6).

### Quantification of viral RNA copy numbers

**Figure 7:**
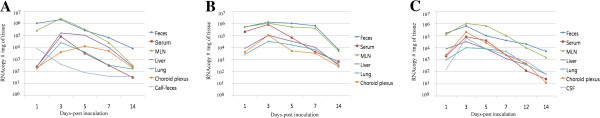
**Quantification of RVA RNA copy numbers by SYBR Green real-time RT-PCR in the feces, serum, mesenteric lymph node (MLN), liver, lung, and choroid plexus. (A)** The sequential changes of viral RNA copy numbers of a porcine G5P[7] strain in the feces, serum, MLN, liver, lung and choroid plexus sampled from virus-infected piglets. Fecal specimens sampled from porcine G5P[7] K71 strain-inoculated calves were also examined by real-time RT-PCR. Note that viral RNA copy numbers in the feces from virus-inoculated calves were markedly lower than those from virus-inoculated piglets. **(B** and **C)** The sequential changes of viral RNA copy numbers in the feces, serum, MLN, liver, lung, and choroid plexus sampled from virus-inoculated piglets **(B)** or calves **(C)** were similar to each other. The geometric means of virus RNA copy numbers per mg of each sample are displayed.

To quantify viral RNA copy numbers in the feces, sera, and extra-intestinal organs and tissues, real-time RT-PCR assays based on SYBR Green chemistry were performed with a primer pair specific to the RVA VP6 gene [39,40,49]. Real-time PCR results showed that viral RNA copy numbers of the porcine RVA strain K71 was observed in the feces of piglet at 1 dpi, reached a peak at 3 dpi, and then gradually declined after 5 dpi (Figure 7A). As expected, viral RNA copy numbers of porcine K71 strain were much lower in the fecal samples of virus-inoculated calves than those of virus-inoculated piglets throughout the experimental period (Figure 7A). These results demonstrate that the porcine K71 strain efficiently replicated and induced pathology in the intestines of piglets, but not in those of calves. The bovine K5 strain replicated and induced pathology in the intestines of both virus-inoculated piglets and calves, but the viral RNA copy numbers were higher in the feces of piglets than in those of the calves, suggesting that strain K5 replicated more efficiently in piglets than in calves (Figure 7B and C).

Viremia and extra-intestinal tropisms have been described in piglets and calves having typical gastrointestinal lesions [50-53]. As described above, the porcine K71 strain was pathogenic only to piglets but the bovine K5 strain was pathogenic to both piglets and calves. Therefore, viremia and extra-intestinal tropisms were evaluated in the sera and extra-intestinal specimens obtained from piglets inoculated with either porcine K71 or bovine K5 strains and calves inoculated only with the K5 strain. The viremia caused by the inoculation of the porcine K71 strain in piglets was detected by nested PCR at 2 dpi, and persisted for 2 or 3 days (Table 1), but no viremia was detected in the calves (Table 3). Real-time PCR results showed that viral RNA in the sera of virus-inoculated piglets were present at 1 dpi, reaching a peak at 3 dpi, and then gradually decreasing (Figure 7A). The viral RNA of bovine K5 strain were detected by nested PCR at 1 or 2 dpi in both virus-inoculated piglets and calves, and lasted to 5 dpi in piglets and to 6 dpi in calves (Tables 2 and 4). Real-time PCR detected viral RNA of bovine K5 strain in the sera from both virus-inoculated piglets and calves at 1 dpi; the copy numbers increased at 3 dpi and then decreased at 5 dpi (Figures 7B and C). Viral RNA copy numbers detected in the sera of piglets were, however, higher than that in calves throughout the whole experimental period (Figures 7B and C). These results indicate that both strains can cause viremia in piglets, but only K5 strain can induce viremia in calves.

Like the sequential changes of virus RNA copy numbers in the feces and sera, the viral copy numbers in the extra-intestinal organs and tissues revealed similar changes in piglets infected with K71 strain and in calves infected with K71 or K5 strains (Figures 7A-C). The viral RNA were detected at 1 dpi, and its copy numbers reached a peak at 3 dpi and then decreased from 5 dpi. Among the extra-intestinal organs and tissues, the MLN from the virus-infected piglet and calves show the highest RNA copy numbers by real-time PCR (Figures 7A-C).

### Comparison of nucleotide and amino acid sequences

Although the bovine K5 and the porcine K71 strains shared the same genotype constellation, their virulence pattern differed between piglets and calves. Therefore, full-length nucleotide and deduced amino acid sequences of all 11 genomes of both strains were compared to each other. Genomic polymorphisms which were found in each of the two strains are listed in Table 6.

**Table 6:** Differences of nucleotide and deduced amino acid sequences between the porcine-like bovine K5 and porcine K71 strains, and between the bovine K5 and other porcine and porcine-like bovine strains.

**Strain**	**Species**	**VP1**		**VP2**		**VP3**	**VP4**	**NSP2**				**NSP3**		**NSP5**
		**R1**		**C1**		**M1**	**P[7]**	**N1**				**E1**		**H1**
		377^a^	2154	830	1063	1845	923	55	522	763	815	64	1032	7
		(120)^b^	(712)	(272)	(349)	(599)	(305)	(3)	(159)	(239)	(257)	(13)	(3*'*UTR)^e^	(5*'*UTR)^f^
K5	Bovine	***G(R)***^c^	T(I)	T(S)	A(L)	***G(S)***	***G(C)***	A(E)	T(F)	T(D)	T(F)	C(S)	***G***	A
K71	Porcine	A(Q)	G(M)	C(P)	C(F)	A(N)	A(Y)	G(E)	C(S)	C(D)	C(L)	T(S)	A	T
PRG942	Porcine	A(Q)	T(I)	C(P)	A(L)	A(N)	–^d^	G(E)	T(F)	T(D)	T(F)	T(S)	A	T
PRG9121	Porcine	A(Q)	T(I)	C(P)	A(L)	A(N)	A(Y)	G(E)	T(F)	C(D)	T(F)	T(S)	A	T
YM	Porcine	A(K)	T(I)	C(P)	A(L)	A(N)	A(Y)	G(E)	T(F)	T(D)	T(F)	A(S)	A	T
Gottfried	Porcine	A(Q)	T(I)	C(P)	A(L)	A(N)	–	G(E)	T(F)	T(D)	T(F)	T(S)	A	A
OSU	Porcine	A(Q)	T(I)	T(S)	A(L)	A(N)	A(Y)	G(E)	T(F)	T(D)	T(F)	T(S)	A	T
A131	Porcine	A(K)	T(I)	–	–	A(N)	A(Y)	G(E)	T(F)	T(D)	T(F)	T(S)	A	T
A253	Porcine	A(K)	T(I)	–	–	A(N)	A(Y)	G(E)	T(F)	T(D)	T(F)	T(S)	A	T
K8	Bovine	* ^g^	*	*	*	*	*	G(E)	T(F)	T(D)	T(F)	T(S)	A	T

^a^ Number of nucleotide site. ^b^ Number of amino acid site. ^c^ Nucleotide (amino acid) changes unique to K5 strain.^d^ Genotype is different from the mentioned above. ^e^ 3′ untraslated region. ^f^ 5′ untraslated region. ^g^ Sequence unavailable.

The comparison of the full-length nucleotide sequences of all 11 genomic segments of bovine K5 and porcine K71 strains identified 13 nucleotide changes. Among them, two nucleotide changes were evident; one in the 3′ untranslated region (UTR) of NSP3 and one in the 5′ UTR of NSP5 (Table 6). The remaining 11 nucleotide changes were observed in the gene coding regions of VP1 (two nucleotides), VP2 (two nucleotides), VP3 (one nucleotide), VP4 (one nucleotide), and NSP2 (four nucleotides) segments. Among the 11 nucleotide changes in the gene coding regions, three were silent mutations, resulting in eight amino acid substitutions (Table 6). Next, we examined if these point mutations were present in other porcine or porcine-like bovine RVA strains, and for strain K5 we found three unique amino acid substitutions in the open reading frames of VP1, VP3 and VP4, along with one nucleotide substitution in the 3′ UTR of the NSP3 segment, which were not present in the other strains examined (Table 6).

## Discussion

The high mutation rate in RVA, the ability of interspecies transmission and genome segments to reassort, and the large pool of RVA in mammals and birds ensure their continuously changing behavior [1]. Knowledge of the virulence and pathogenicity of RVA strains in different host species is key to understanding methods of prevention and control of RVA infections. Direct interspecies transmissions of RVA have been reported to occur in nature on a number of occasions [31,32,54]. However, the pathogenicity of such strains remains largely unknown in different host species. In our previous report [32], the bovine K5 strain has been isolated from a diarrheic calf, and characterized as a porcine-like RVA strain due to having the same genotype constellation as the porcine RVA reference strain OSU. In the present study, we further characterized its pathogenicity in its homologous (piglets) and heterologous (calves) hosts. In addition, the virulence of the bovine K5 strain in the homologous and heterologous hosts was compared with that of the porcine K71 strain. The porcine K71 strain was isolated from a diarrheic piglet and was identified as being an OSU-like strain with a genotype constellation identical to that of the OSU strain [11]. On the contrary to our expectations, the bovine K5 strain showed pronounced virulence and pathogenicity in the homologous (piglets) as well as heterologous (calves) hosts; piglets and calves infected with this strain had continuous diarrhea, high virus RNA copy numbers in the fecal samples, and severe and large histopathological lesions in the small intestinal mucosa. As expected, however, the porcine K71 strain had severe virulence and pathogenicity in piglets, but not in calves. When RVA cross the host species barrier, this is usually a dead-end infection since the virus is not able to properly spread in the new host species. However, if the interspecies transmitted virus is able to reassort with RVA inherent in that host species, the resultant reassortant strains may have a higher probability to efficiently infect and spread among the population of the new host [5,25,54,55]. On the contrary to this general belief, our results highlight the existence of different enteropathogenic virulence depending on the strains, even among those with the same genotype constellation in nature. To our knowledge, this is the first study on the virulence and host range restriction of a particular RVA strain in its homologous and heterologous hosts.

Although the virulence and pathogenicity of heterologous RVA infections have never been documented under natural conditions, they have been reported in experimental conditions. Simian RVA strain RRV and lapine strains share similar VP4, VP7 and NSP1, which have been implicated in host range restriction, and the simian RRV strain has been shown to replicate efficiently and to transmit horizontally in a rabbit model [56]. Therefore, it is possible that the possession of these three related genes may confer RRV the ability to replicate efficiently in rabbits [56]. Genetically, human DS-1-like strains are believed to have a common origin with bovine RVA strains because they share the same genotypes of six genomic segments (VP6, VP1, VP2, VP3, NSP2, and NSP4) [5]. Moreover, DS-1-like strains have been shown to successfully infect cattle [57,58], but not piglets [26]. On the contrary, the virulent human RVA strain Wa has a similar genomic constellation to those of porcine RVA, i.e., only the VP7, VP4, and VP6 genotypes are different with the porcine RVA strain OSU, VP7, VP4, and NSP1 with the porcine RVA strain Gottfried, and VP7, VP4, VP6, and VP2 genes with the porcine RVA strain A131 [5]. Moreover, the human RVA strain Wa induces diarrhea and pathology in the small intestine of piglets [38,59]. Based on these genomic and pathological properties, it has been speculated that human Wa-like RVA strains could have a common origin with the porcine Wa-like strains [5]. In the present study, both the bovine K5 and the porcine K71 RVA strains shared the genotype constellation with porcine RVA strain OSU, and induced diarrhea and intestinal pathology in piglets. In calves, however, the virulence of both strains was different; the bovine K5 strain induced diarrhea and intestinal pathology, but the porcine K71 did not. It is still unknown why the genetically nearly identical porcine K71 and bovine K5 strains had different virulence in calves. Interestingly, the human Wa-like strain D can also infect and induce mild disease in calves [5,57]. Like the human Wa-like strain D, the bovine K5 strain showed virulence not only in piglets, but also in calves.

Host specificity and pathogenicity of RVA may be the result of the interaction of numerous host factors and viral proteins. However, the molecular basis for virulence and/or host range restriction of the RVA strain is still unclear. To date, a number of gene segments have shown to be capable of affecting host range restriction and/or virulence (VP3, VP4, VP7, NSP1, NSP2, and NSP4) [22-28]. Most of these experiments were conducted using reassortant strains possessing quite different parental viruses. Our data show highly different virulence profiles in different hosts between two closely related RVA strains with identical genotype constellations. This indicates that differences in virulence and host range restriction cannot be fully explained by the genotype constellation.

The present sequence comparison between the bovine K5 and porcine K71 strains revealed that the bovine K5 strain harbored two nucleotide differences in the 5′ and 3′ UTR, and 11 nucleotide differences in the gene coding regions, resulting in eight amino acid substitutions. These unique mutations between the two strains may be possible candidates for virulence determinants in the heterologous host (Table 6). Since little information is available about the key virulence determinants (mutations) responsible for virulence and pathogenicity of RVA in a heterologous host, it is still uncertain whether the increased virulence and pathogenicity of bovine K5 strain in the heterologous host (calves) is induced by any single substitution in a certain gene or by a combination of these substitutions. Furthermore, three amino acid substitutions in VP1, VP3 and VP4, and one nucleotide substitution in the 3′ UTR of K5 were found to be unique in comparison with other porcine and bovine RVA reference strains. Since the virulence and pathogenicity in the heterologous and/or homologous hosts of these porcine and porcine-like bovine RVA reference strains has not been tested, it is still uncertain whether the increased virulence and pathogenicity of bovine K5 strain in the heterologous host (calves) are induced by the observed substitutions. Therefore, the molecular and biological properties of each genome segment, and its possible association with host range restriction and/or virulence will be needed to be clarified in further studies, using reverse genetics to swap individual genes of these viruses or introduce particular mutations. The exact mechanisms of how the bovine K5 strain acquired these specific mutations are unknown. The mutations of strain K5 could have been acquired by serial replication in vivo in calves, or alternatively, the mutations of strain K5 could have occurred during serial replication in vivo in piglets, and subsequently have been transmitted to calves.

In human RVA infections, RVA antigen and viral RNA have been sporadically detected in the serum and various extra-intestinal organs and tissues [34,60,61]. Moreover, viremia and extra-intestinal pathogenicity of RVA infection have been clearly demonstrated in experimental animal models [35-37,39,40,62]. However, little information is available as to whether heterologous RVA strains cause viremia and extra-intestinal pathogenicity in heterologous hosts. To address this point, we sequentially collected serum and extra-intestinal organs and tissues from piglets and calves inoculated with the bovine heterologous strain K5. Both piglets and calves inoculated with the K5 strain showed similar dynamics of viral RNA in the sera and extra-intestinal organs and tissues. Viral RNA was detected at 1 dpi and its concentration reached a peak at 3 or 5 dpi, and then gradually decreased. Viral antigen was detected in these extra-intestinal organs and tissues where degeneration and/or necrosis of the corresponding parenchymal cells were observed. Although this is the first report that the heterologous RVA strain causes viremia and extra-intestinal pathogenicity in its heterologous hosts, the patterns of viremia and extra-intestinal infection are consistent with those of other reports [35-37,39,40,62].

In conclusion, bovine heterologous K5 strain, despite having the same genomic constellation with the porcine G5P[7] prototype OSU and the Korean porcine K71 strains, displays pronounced virulence, and intestinal and extra-intestinal pathogenicities in a heterologous host. In addition, non-synonymous mutations of bovine K5 strain compared with those of porcine homologous K71 strain may contribute to virus replication ability, virulence, and pathogenicity in the heterologous host (calves). These preliminary observations contribute to our understanding of RVA infection in homologous and heterologous hosts. To further clarify the molecular and biological properties of each genomic segment associated with host range restriction and/or virulence, a combination of standard biochemical and molecular tools, together with the experimental inoculation studies with many heterologous strains in their natural homologous and heterologous hosts and new technologies such as reverse genetics system, will be required.

## Competing interests

The authors declare that they have no competing interests.

## Authors’ contributions

JGP and HJK carried out the all of animal experimental procedures, IFA, sequencing, RT-PCR, nested PCR, real time RT-PCR, histopathological experiment, and drafted the manuscript. JM made substantial contributions to the analysis of genomic characterization and helped to revise and draft the manuscript. JGP and MMA were involved in RACE PCR. DSK, MH, and JYK participated in the IFA, RT-PCR, and nested PCR. RBC, KYS, and HER contributed to the histopathological experiment. MIK and SIP participated in the design of the study and in the evaluation of histopathology. KOC conceived the experimental design, participated in its coordination and helped to interpret data and draft the manuscript. All authors have read and approved the final manuscript.

## Supplementary Material

Additional file 1 **Oligonucleotide primers for sequencing or for 5′ and 3′ RACE PCR of all eleven genomic segments of the porcine K71 and bovine K5 rotavirus strains.** The primer pairs used to generate the full-length sequence of porcine K71 and bovine K5 rotavirus strains are listed in the table. Also indicated are the gene-specific primers used for 5′ and 3′ RACE PCR. Click here for file

Additional file 2 **Genbank accession numbers and nucleotide sequence identities (%) of open reading frames in each gene segment of the G5P[7] (K71 and K5) Korean porcine rotavirus strains to those of other known rotaviruses.** The nucleotide sequences of open reading frame of the porcine K71 and bovine K5 rotavirus strains were compared with known RVA strains. The values represent the nucleotide similarity of porcine K71 and bovine K5 with the reference strains. Click here for file

Additional file 3 **Comparison of genotype constellation of porcine and porcine-like G5P[7] strains with other known reference genotypes.** The proposed classification by the RCWG was applied to the structural and non-structural protein encoding genes for porcine K71 and bovine K5. Green was used for Wa-like strains, while red indicates DS-1 like strains. Blue and dark green were used for some typical porcine segments, while purple and pink were used for bovine-like segments. Click here for file

Additional file 4 **Phylogenentic trees based on the full length nucleotide sequence of the 11 genomic segments of porcine G5P[7] K71 and porcine-like bovine G5P[7] K5 strains in comparison with reference RVA strains.** The full length sequence of the 11 segments of porcine G5P[7] K71 and porcine-like bovine G5P[7] K5 strains were aligned, and phylogenetic trees were constructed using the neighbor-joining method with 1000 bootstrap replicates. Genetic distances were calculated using Kimura-2 correction parameter at the nucleotide level. Click here for file

Additional file 5 **Summary of the histopathological findings in the small intestine of the colostrum-deprived piglets inoculated with a G5P[7] K71 strain.** Histopathological lesions of the small intestine were scored according to the average villi/crypt (V/C) ratio plus the grade of epithelial cell desquamation. The lesion score for the small intestine was calculated by measuring 10 randomly selected villi and crypts. The number of antigen-positive cells in the villi was determined using indirect immunofluorescence assay with monoclonal antibody against the VP6 protein of strain OSU. Click here for file

Additional file 6 **Summary of the histopathological findings in the small intestine of the colostrum-deprived calves inoculated with a porcine G5P[7] K71 strain.** The average villi/crypt (V/C) ratio plus the grade of epithelial cell desquamation of 10 randomly selected villi and crypt were used to determine the histopathological lesion in the small intestine of colostrum-deprived calves inoculated with porcine K71 strain. Indirect immunofluorescence assay with monoclonal antibody against the VP6 protein of strain OSU was conducted to measure the antigen distribution in the small intestine. Click here for file

Additional file 7 **Summary of the histopathological findings in the small intestine of the colostrum-deprived piglets inoculated with a bovine G5P[7] K5 strain.** The small intestinal changes were scored according to the average villi/crypt (V/C) ratio plus the grade of epithelial cell desquamation. The antigen distribution in the small intestine was evaluated based on the number of antigen-positive cells in the villi using an indirect immunofluorescence assay with monoclonal antibody against the VP6 protein of strain OSU. Click here for file

Additional file 8 **Summary of the histopathological findings in the small intestine of the colostrum-deprived calves inoculated with a bovine G5P[7] K5 strain.** The average villi/crypt (V/C) ratio plus the grade of epithelial cell desquamation of 10 randomly selected villi and crypt were used to determine the histopathological lesion in the small intestine of colostrum-deprived calves inoculated with bovine K5 strain. Indirect immunofluorescence assay with monoclonal antibody against the VP6 protein of strain OSU was conducted to measure the antigen distribution in the small intestine. Click here for file

Additional file 9 **Summary of the antigen distribution in the extraintestinal organs of the colostrum-deprived piglets inoculated with a porcine G5P[7] K71 strain.** To determine the antigen distribution in the extraintestinal organs, the number of antigen-positive cells was evaluated using the indirect immunofluorescence assay with monoclonal antibody against the VP6 protein of strain OSU. Values represent the average that was calculated in 10 fields per section. Click here for file

Additional file 10 **Summary of the antigen distribution in the extraintestinal organs of the colostrum-deprived calves inoculated with a porcine G5P[7] K71 strain.** Major organs such as the lung, liver, mesenteric lymph node, and choroid plexus were subjected to indirect immunofluorescence assay with monoclonal antibody against the VP6 protein of strain OSU to determine if RVA antigen-positive cells were present. Ten fields per section were analyzed to calculate the average number of antigen-positive cells. Click here for file

Additional file 11 **Summary of the antigen distribution in the extraintestinal organs of the colostrum-deprived piglets inoculated with a bovine G5P[7] K5 strain.** The antigen distribution in the extraintestinal organs was evaluated based on the number of antigen-positive cells. To calculate the number positive cells in the organs or tissues, 10 fields per section were analyzed with indirect immunofluorescence assay with monoclonal antibody against the VP6 protein of strain OSU. Click here for file

Additional file 12 **Summary of the antigen distribution in the extraintestinal organs of the colostrum-deprived calves after inoculation with a bovine G5P[7] K5 strain.** The data represent the average value of antigen-positive cells in the mesenteric lymph nodes, livers, lungs, and choroid plexuses of calves inoculated with bovine K5 strain. Ten fields per section were analyzed to calculate the average number of antigen-positive cells with indirect immunofluorescence assay with monoclonal antibody against the VP6 protein of strain OSU. Click here for file
