# Supplementary material for: Different virulence of porcine and porcine-like bovine rotavirus strains with genetically nearly identical genomes in piglets and calves
Source: Vet Res. 2013 Oct 1;44(1):88. doi: 10.1186/1297-9716-44-88 (PMC3851489; doi:10.1186/1297-9716-44-88)
Supplement: Additional file 1 — Oligonucleotide primers for sequencing or for 5′ and 3′ RACE PCR of all eleven genomic segments of the porcine K71 and bovine K5 rotavirus strains. The primer pairs used to generate the full-length sequence of porcine K71 and bovine K5 rotavirus strains are listed in the table. Also indicated are the gene-specific primers used for 5′ and 3′ RACE PCR. [file 1297-9716-44-88-S1.docx]

**Supplementary Table 1 Oligonucleotide primers for sequencing or for 5’ and 3’ RACE PCRs of all eleven genomic segments of the porcine K71 and bovine K5 rotavirus strains.**

| Target  gene | Primer name | Sequence (5’-3’) ^a^ | Region  (nt) | Size  (bp) | References |
| --- | --- | --- | --- | --- | --- |
| VP1 | GEN-VP1F | F:GGCTATTAAAGCTRTACAATGGGGAAG | 1–27 | 1592 | [5] |
|  |  | R:TCC CAC TGG GAM ACG TCT GTA TAT | 1569–1592 |  | [20] |
|  |  | F:GAATTCTAC TCACAGTCAA AT | 1492–1512 | 1811 | [20]  [20] |
|  |  | R:GGTCACATCTAAGCGCTCTAATCTTS | 3281–3302 |  |  |
|  | VP1-5’ ^b^ | R:CGAGTTCATTCGCCGTCAAATCTGCTTC | 340-313 | 340 | In this study |
|  | VP1-3’ ^c^ | F: TTTCACTAGGAGTCCCACCAGTTGATGC | 2906-2933 | 396 | In this study |
| VP2 |  | F:GGCTATTRAAGGYTCAATGGCGTACAG | 1–27 | 1393 | [20]  [20] |
|  |  | R:TTTCTATAATGCATTCKTTGCATT | 1375–1393 |  |  |
|  |  | F:ATAAAYTCACA AGCAGCAAAT GA | 1160–1182 | 1558 | [20] |
|  | GEN-VP2_Rbc | R:GTCATATCTCCACARTGGGGTTGG | 2666–2689 |  | [5] |
|  | VP2-5’ | R: CATAATCTCCATCTGGCAGCGTGTCTCT | 521-494 | 521 | In this study |
|  | VP2-3’ | F:CTGGATGCTACAGTTTTCGCCCAGATAG | 2378-2405 | 369 | In this study |
| VP3 |  | F:GGCTTTTTAAAGCAATATTAGTA | 1–23 | 970 | [20]  [20] |
|  |  | R:ATGGTGTGTCCAATGGATCC | 970–989 |  |  |
|  |  | F:GGATCCATTGGACACACCAT | 970–989 | 1603 | [20]  [20] |
|  |  | R:GGTCACGACCTGACCATGGTG | 2576–2596 |  |  |
|  | VP3-5’ | R:ATTATAGTGACCTCGCGTGTCCTTTCCA | 466-439 | 466 | In this study |
|  | VP3-3’ | F:GATCAGCCAAAGAGTTTGCTGCGTTG | 2028-2053 | 563 | In this study |
| VP4 | GEN-VP4F | F:GGCTATAAAATGGCTTCGCTCA | 1–22 | 868 | [5] |
|  |  | R:ATTTCGGACCATTTATAA CC | 868–887 |  | [43] |
|  |  | F:GGT TAT AAA TGG TCC GAA AT | 868–887 | 1477 | [20]  [20] |
|  |  | R:GGYCWCAACCTCTAGACACT | 2343–2362 |  |  |
|  | VP4-5’ | R:CCCTCTACAGTTGGCGCAAGTAGTACCC- | 278-251 | 278 | In this study |
|  | VP4-3’ | F:CAATAGGATCATCAGCATCCGCTTGGAC | 1757-1784 | 605 | In this study |
| VP6 | GEN-VP6F | F:GGCTTTWAAACGAAGTCTTC | 1–20 | 1356 | [5]  [5] |
|  | GEN-VP6R | R:GGTCACATCCTCTCACT | 1340–1356 |  |  |
|  | VP6-5’ | R:GAGCTATTCCGTTTCGTTGCGACTCTCT | 354-327 | 354 | In this study |
|  | VP6-3’ | F:GTGTTCCCACCAGGTATGAATTGGACAG | 1101-1128 | 255 | In this study |
| VP7 |  | F:GCCTTTAAAAGCGAGAATTT | 1–20 | 1062 | [44]  [44] |
|  |  | R:GGTCACATCATACAACTCTA | 1043–1062 |  |  |
|  | VP7-5’ | R:GAGCTTTAATGAGCGGTGCAAGTACGAC | 199-172 | 199 | In this study |
|  | VP7-3’ | F:TGTCGCTGTAATTCAGGTAGGAGGTCCA | 819-846 | 243 | In this study |
| NSP1 | LAP-NSP1-F | F:GGGCTTTTTTTTGAAAAGTC | 1–20 | 1567 | [30]  [46] |
|  | VF5R | R:GGTCACATTTTATGCTGCCTA | 1547–1567 |  |  |
|  | NSP1-5’ | R:CATGATACATGGTACAGCCTCGACAG | 215-190 | 215 | In this study |
|  | NSP1-3’ | F:GCCACTGAGGTACACAACTGCAAATGG | 974-1000 | 592 | In this study |
| NSP2 | VF3F | F:GGCTTTTAAAGCGTCTCAGTC | 1–21 | 1058 | [46]  [46] |
|  | VF3R | R:GGTCACATAAGCGCTTTCTATTC | 1036–1058 |  |  |
|  | NSP2-5’ | R:CTTCAGCAGTGGCAGTGGTTTCAATTTC | 506-479 | 506 | In this study |
|  | NSP2-3’ | F:CACGCAGACAGAGTATTCGCTACA | 755-778 | 304 | In this study |
| NSP3 | VF2F | F:ATGCTCAAGATGGAGTCTACT | 1–21 | 1050 | [46]  [46] |
|  | VF2R | R:GGTCACATAACGCCCCTATAG | 1030–1050 |  |  |
|  | NSP3-5’ | R:AGTGCCTGATCAATAGTCGCAGCTTTGC | 246-219 | 246 | In this study |
|  | NSP3-3’ | F:GAGGTCCATGGAATTGTCAGATGATGTC | 772-799 | 303 | In this study |
| NSP4 | 10Beg16 | F:TGTTCCGAGAGAGCGCGTG | 16–34 | 725 | [45]  [45] |
|  | 10End722c | R:GACCATTCCTTCCATTAAC | 722–740 |  |  |
|  | NSP4-5’ | R:CGAACACTTCGACGTTCTCAACGCTATT | 233-206 | 233 | In this study |
|  | NSP4-3’ | F:CTATGTGAGAGGTTGAGTTGCCGTCGTC | 562-589 | 189 | In this study |
| NSP5 | VF1F | F:GGCTTTTAAAGCGCTACAGTG | 1–21 | 664 | [46]  [46] |
|  | VF1R | R:GGTCACAAAACGGGAGTGGG | 645–664 |  |  |
|  | NSP5-5’ | R:GATCGCACCCAACGTTACTTGAAGGTC | 340-314 | 340 | In this study |
|  | NSP5-3’ | F:GTGCGATCAAGTGGATTTCTCCCTGACT | 333-360 | 331 | In this study |

**^a^**F, forward; R, reverse.

^b^Primer for 5’ RACE PCR.

^c^Primer for 3’ RACE PCR.
