# Supplementary material for: Different virulence of porcine and porcine-like bovine rotavirus strains with genetically nearly identical genomes in piglets and calves
Source: Vet Res. 2013 Oct 1;44(1):88. doi: 10.1186/1297-9716-44-88 (PMC3851489; doi:10.1186/1297-9716-44-88)
Supplement: Additional file 2 — Genbank accession numbers and nucleotide sequence identities (%) of open reading frames in each gene segment of the G5P[7] (K71 and K5) Korean porcine rotavirus strains to those of other known rotaviruses. The nucleotide sequences of open reading frame of the porcine K71 and bovine K5 rotavirus strains were compared with known RVA strains. The values represent the nucleotide similarity of porcine K71 and bovine K5 with the reference strains. [file 1297-9716-44-88-S2.docx]

**Additional file 2 Genbank accession numbers and nucleotide sequence identities (%) of open reading frame in each gene segment of the G5P[7] (K71 and K5) Korean porcine rotavirus strains to those of other known rotaviruses.**

|  | Genotype^a^ | Strain^b^ | Accession Number | % nt identity with strains^b^ | |
| --- | --- | --- | --- | --- | --- |
| Segment |  |  |  | K5 | K71 |
| VP7 | G1 | RVA/Human-tc/USA/Wa/1974/G1P1A[8] | M21843 | 65.50 | 65.50 |
|  | G1 | RVA/Panda-tc/CHN/CH-1/2008/G1P[7] | GU188284 | 65.60 | 65.60 |
|  | G2 | RVA/Human-tc/USA/DS-1/1976/G2P1B[4] | EF672581 | 57.70 | 57.70 |
|  | G3 | RVA/Pig-tc/VEN/A131/1988/G3P9[7] | L35055 | 69.10 | 69.10 |
|  | G4 | RVA/Pig-tc/USA/Gottfried/1983/G4P[6] | X06759 | 61.10 | 61.10 |
|  | **G5** | **RVA/Cow-tc/KOR/K5/2004/G5P[7]** | **JX971574** | **-** | **100.00** |
|  | **G5** | **RVA/Pig-tc/KOR/K71/2006/G5P[7]** | **JX971568** | **100.00** | **-** |
|  | **G5** | **RVA/Cow-tc/KOR/K8/2005/G5P[7]** | **EU541405** | **99.80** | **99.80** |
|  | **G5** | **RVA/Pig-tc/USA/OSU/1977/G5P9[7]** | **X04613** | **99.90** | **99.90** |
|  | **G5** | **RVA/Horse-tc/GBR/H-1/1975/G5P[7]** | **AF242393** | **96.50** | **96.50** |
|  | **G5** | **RVA/Pig-xx/CHN/JL94/XXXX/G5P[7]** | **AY538665** | **99.90** | **99.90** |
|  | **G5** | **RVA/Cow-tc/KOR/KV0407/2004/G5P[7]** | **EU873013** | **100.00** | **100.00** |
|  | **G5** | **RVA/Pig-tc/KOR/B-1/2006/G5P[7]** | **FJ807851** | **100.00** | **100.00** |
|  | **G5** | **RVA/Pig-tc/KOR/150-1/2006/G5P[7]** | **FJ807847** | **100.00** | **100.00** |
|  | **G5** | **RVA/Pig-tc/KOR/122-1/2006/G5P[7]** | **FJ807844** | **100.00** | **100.00** |
|  | G6 | RVA/Cow-tc/KOR/KJ9-1/2006/G6P[7] | FJ206034 | 60.80 | 60.80 |
|  | G8 | RVA/Cow-tc/KOR/KJ25-1/2006/G8P[7] | FJ206044 | 66.70 | 66.70 |
|  | G8 | RVA/Cow-tc/KOR/KJ246/2006/G8P[7] | FJ206084 | 66.50 | 66.50 |
|  | G8 | RVA/Cow-tc/KOR/KJ330-1/2006/G8P[7] | FJ206087 | 65.70 | 65.70 |
|  | G8 | RVA/Cow-tc/KOR/KJ338-1/2006/G8P[7] | FJ206088 | 66.80 | 66.80 |
|  | G9 | RVA/Pig-tc/KOR/PRG9121/2006/G9P[7] | JF796739 | 76.45 | 76.45 |
|  | G9 | RVA/Pig-tc/KOR/PRG942/2006/G9P[23] | JF796728 | 76.25 | 76.25 |
|  | G11 | RVA/Pig-tc/MEX/YM/1983/G11P9[7] | M23194 | 77.30 | 77.30 |
|  | G11 | RVA/Pig-tc/VEN/A253/1988/G11P9[7] | L24163 | 81.10 | 81.10 |
| VP4 | P[4] | RVA/Human-tc/USA/DS-1/1976/G2P1B[4] | EF672577 | 45.36 | 45.45 |
|  | P[6] | RVA/Pig-tc/USA/Gottfried/1983/G4P[6] | M33516 | 44.76 | 44.85 |
|  | **P[7]** | **RVA/Cow-tc/KOR/K5/2004/G5P[7]** | **JX971572** | **-** | **99.96** |
|  | **P[7]** | **RVA/Pig-tc/KOR/K71/2006/G5P[7]** | **JX971567** | **99.96** | **-** |
|  | **P[7]** | **RVA/Panda-tc/CHN/CH-1/2008/G1P[7]** | **HQ641296** | **99.44** | **99.48** |
|  | **P[7]** | **RVA/Pig-tc/USA/OSU/1977/G5P9[7]** | **X13190** | **99.48** | **99.52** |
|  | **P[7]** | **RVA/Horse-tc/GBR/H-1/1975/G5P[7]** | **FJ870377** | **93.82** | **93.87** |
|  | **P[7]** | **RVA/Pig-xx/CHN/JL94/XXXX/G5P[7]** | **AY523636** | **99.44** | **99.48** |
|  | **P[7]** | **RVA/Cow-tc/KOR/KV0407/2004/G5P[7]** | **EU873009** | **99.65** | **99.70** |
|  | **P[7]** | **RVA/Cow-tc/KOR/KJ9-1/2006/G6P[7]** | **HM988970** | **99.26** | **99.31** |
|  | **P[7]** | **RVA/Cow-tc/KOR/KJ25-1/2006/G8P[7]** | **HM988968** | **99.48** | **99.52** |
|  | **P[7]** | **RVA/Pig-tc/VEN/A131/1988/G3P9[7]** | **None^c^** | **92.51** | **92.56** |
|  | **P[7]** | **RVA/Pig-tc/VEN/A253/1988/G11P9[7]** | **None** | **92.77** | **92.82** |
|  | **P[7]** | **RVA/Pig-tc/MEX/YM/1983/G11P9[7]** | **M63231** | **91.82** | **91.86** |
|  | **P[7]** | **RVA/Pig-tc/KOR/PRG9121/2006/G9P[7]** | **JF796737** | **92.67** | **92.71** |
|  | P[8] | RVA/Human-tc/USA/Wa/1974/G1P1A[8] | L34161 | 42.29 | 42.38 |
|  | P[23] | RVA/Pig-tc/KOR/PRG942/2006/G9P[23] | JF796726 | 73.93 | 73.97 |
| VP6 | I1 | RVA/Human-tc/USA/Wa/1974/G1P1A[8] | K02086 | 76.48 | 76.48 |
|  | I1 | RVA/Pig-tc/USA/Gottfried/1983/G4P[6] | D00326 | 76.98 | 76.98 |
|  | I2 | RVA/Human-tc/USA/DS-1/1976/G2P1B[4] | HQ650121 | 70.37 | 70.37 |
|  | I2 | RVA/Cow-tc/KOR/KJ9-1/2006/G6P[7] | HM988974 | 71.94 | 71.94 |
|  | **I5** | **RVA/Cow-tc/KOR/K5/2004/G5P[7]** | **JX971573** | **-** | **100.00** |
|  | **I5** | **RVA/Pig-tc/KOR/K71/2006/G5P[7]** | **JX971583** | **100.00** | **-** |
|  | **I5** | **RVA/Pig-tc/VEN/A131/1988/G3P9[7]** | **AF317124** | **85.09** | **85.09** |
|  | **I5** | **RVA/Pig-tc/USA/OSU/1977/G5P9[7]** | **AF317123** | **84.02** | **84.02** |
|  | **I5** | **RVA/Horse-tc/GBR/H-1/1975/G5P[7]** | **AF242394** | **85.35** | **85.35** |
|  | **I5** | **RVA/Pig-xx/CHN/JL94/XXXX/G5P[7]** | **AY538664** | **99.66** | **99.66** |
|  | **I5** | **RVA/Cow-tc/KOR/KV0407/2004/G5P[7]** | **EU873010** | **100.00** | **100.00** |
|  | **I5** | **RVA/Panda-tc/CHN/CH-1/2008/G1P[7]** | **GU188283** | **99.16** | **99.16** |
|  | **I5** | **RVA/Cow-tc/KOR/KJ25-1/2006/G8P[7]** | **HM988972** | **88.70** | **88.70** |
|  | **I5** | **RVA/Pig-tc/VEN/A253/1988/G11P9[7]** | **AF317122** | **83.86** | **83.86** |
|  | **I5** | **RVA/Pig-tc/MEX/YM/1983/G11P9[7]** | **X69487** | **87.68** | **87.68** |
| VP1 | **R1** | **RVA/Cow-tc/KOR/K5/2004/G5P[7]** | **JX971569** | **-** | **99.94** |
|  | **R1** | **RVA/Pig-tc/KOR/K71/2006/G5P[7]** | **JX971580** | **99.94** | **-** |
|  | **R1** | **RVA/Human-tc/USA/Wa/1974/G1P1A[8]** | **DQ490539** | **84.89** | **84.89** |
|  | **R1** | **RVA/Pig-tc/USA/OSU/1977/G5P9[7]** | **GU199514** | **99.69** | **99.69** |
|  | **R1** | **RVA/Horse-tc/GBR/H-1/1975/G5P[7]** | **JQ309138** | **85.20** | **85.19** |
|  | **R1** | **RVA/Panda-tc/CHN/CH-1/2008/G1P[7]** | **HQ641297** | **99.75** | **99.75** |
|  | **R1** | **RVA/Cow-tc/KOR/KJ25-1/2006/G8P[7]** | **HM988965** | **95.27** | **95.27** |
|  | **R1** | **RVA/Pig-tc/VEN/A131/1988/G3P9[7]** | **EF560618** | **83.60** | **83.59** |
|  | **R1** | **RVA/Pig-tc/VEN/A253/1988/G11P9[7]** | **EF560621** | **83.27** | **83.27** |
|  | **R1** | **RVA/Pig-tc/MEX/YM/1983/G11P9[7]** | **X76486** | **86.14** | **86.14** |
|  | **R1** | **RVA/Pig-tc/USA/Gottfried/1983/G4P[6]** | **M32805** | **96.16** | **96.16** |
|  | **R1** | **RVA/Pig-tc/KOR/PRG942/2006/G9P[23]** | **JF796723** | **86.01** | **86.01** |
|  | **R1** | **RVA/Pig-tc/KOR/PRG9121/2006/G9P[7]** | **JF796734** | **86.16** | **86.16** |
|  | R2 | RVA/Human-tc/USA/DS-1/1976/G2P1B[4] | EF990691 | 73.08 | 73.03 |
|  | R2 | RVA/Cow-tc/KOR/KJ9-1/2006/G6P[7] | HM988967 | 72.70 | 72.69 |
| VP2 | **C1** | **RVA/Cow-tc/KOR/K5/2004/G5P[7]** | **JX971570** | **-** | **99.92** |
|  | **C1** | **RVA/Pig-tc/KOR/K71/2006/G5P[7]** | **JX971581** | **99.92** | **-** |
|  | **C1** | **RVA/Human-tc/USA/Wa/1974/G1P1A[8]** | **X14942** | **86.86** | **86.85** |
|  | **C1** | **RVA/Panda-tc/CHN/CH-1/2008/G1P[7]** | **HQ641294** | **84.31** | **84.36** |
|  | **C1** | **RVA/Horse-tc/GBR/H-1/1975/G5P[7]** | **JQ309139** | **96.49** | **96.49** |
|  | **C1** | **RVA/Cow-tc/KOR/KJ9-1/2006/G6P[7]** | **HM988960** | **89.27** | **89.27** |
|  | **C1** | **RVA/Cow-tc/KOR/KJ25-1/2006/G8P[7]** | **HM988959** | **89.40** | **89.40** |
|  | **C1** | **RVA/Pig-tc/USA/Gottfried/1983/G4P[6]** | **GU199487** | **93.28** | **93.28** |
|  | **C1** | **RVA/Pig-tc/USA/OSU/1977/G5P9[7]** | **GU199515** | **99.96** | **99.86** |
|  | **C1** | **RVA/Pig-tc/MEX/YM/1983/G11P9[7]** | **GU199516** | **93.56** | **93.56** |
|  | **C1** | **RVA/Pig-tc/KOR/PRG942/2006/G9P[23]** | **JF796724** | **92.48** | **92.48** |
|  | **C1** | **RVA/Pig-tc/KOR/PRG9121/2006/G9P[7]** | **JF796735** | **93.60** | **93.60** |
|  | C2 | RVA/Human-tc/USA/DS-1/1976/G2P1B[4] | HQ650117 | 69.20 | 69.17 |
|  | C2 | RVA/Pig-tc/VEN/A131/1988/G3P9[7] | EF560619 | 59.91 | 59.86 |
|  | C2 | RVA/Pig-tc/VEN/A253/1988/G11P9[7] | EF560622 | 62.28 | 62.23 |
| VP3 | **M1** | **RVA/Cow-tc/KOR/K5/2004/G5P[7]** | **JX971571** | **-** | **99.96** |
|  | **M1** | **RVA/Pig-tc/KOR/K71/2006/G5P[7]** | **JX971582** | **99.96** | **-** |
|  | **M1** | **RVA/Human-tc/USA/Wa/1974/G1P1A[8]** | **AY267335** | **87.91** | **87.96** |
|  | **M1** | **RVA/Panda-tc/CHN/CH-1/2008/G1P[7]** | **HQ641295** | **83.40** | **83.45** |
|  | **M1** | **RVA/Pig-tc/USA/Gottfried/1983/G4P[6]** | **GU199488** | **88.37** | **88.42** |
|  | **M1** | **RVA/Pig-tc/USA/OSU/1977/G5P9[7]** | **AY277921** | **99.64** | **99.68** |
|  | **M1** | **RVA/Horse-tc/GBR/H-1/1975/G5P[7]** | **JQ309140** | **92.13** | **92.17** |
|  | **M1** | **RVA/Pig-tc/VEN/A131/1988/G3P9[7]** | **EF560620** | **96.35** | **96.39** |
|  | **M1** | **RVA/Pig-tc/VEN/A253/1988/G11P9[7]** | **EF560623** | **96.21** | **96.25** |
|  | **M1** | **RVA/Pig-tc/MEX/YM/1983/G11P9[7]** | **AY300922** | **91.54** | **91.59** |
|  | **M1** | **RVA/Pig-tc/KOR/PRG942/2006/G9P[23]** | **JF796725** | **86.76** | **86.80** |
|  | **M1** | **RVA/Pig-tc/KOR/PRG9121/2006/G9P[7]** | **JF796736** | **88.08** | **88.12** |
|  | M2 | RVA/Human-tc/USA/DS-1/1976/G2P1B[4] | EF990693 | 56.28 | 56.39 |
|  | M2 | RVA/Cow-tc/KOR/KJ9-1/2006/G6P[7] | HM988964 | 54.65 | 54.77 |
|  | M2 | RVA/Cow-tc/KOR/KJ25-1/2006/G8P[7] | HM988962 | 55.20 | 55.32 |
| NSP1 | **A1** | **RVA/Cow-tc/KOR/K5/2004/G5P[7]** | **JX971575** | **-** | **100.00** |
|  | **A1** | **RVA/Pig-tc/KOR/K71/2006/G5P[7]** | **JX971584** | **100.00** | **-** |
|  | **A1** | **RVA/Human-tc/USA/Wa/1974/G1P1A[8]** | **JX406751** | **83.15** | **83.15** |
|  | **A1** | **RVA/Panda-tc/CHN/CH-1/2008/G1P[7]** | **GU205762** | **99.93** | **99.93** |
|  | **A1** | **RVA/Pig-tc/USA/OSU/1977/G5P9[7]** | **U08432** | **98.81** | **98.81** |
|  | **A1** | **RVA/Cow-tc/KOR/KJ9-1/2006/G6P[7]** | **FJ206224** | **99.58** | **99.58** |
|  | **A1** | **RVA/Cow-tc/KOR/KJ25-1/2006/G8P[7]** | **FJ206198** | **100.00** | **100.00** |
|  | **A1** | **RVA/Pig-tc/VEN/A253/1988/G11P9[7]** | **EF990695** | **95.35** | **95.35** |
|  | **A1** | **RVA/Pig-tc/VEN/A131/1988/G3P9[7]** | **EF990687** | **92.99** | **92.99** |
|  | **A1** | **RVA/Cow-tc/KOR/K8/2005/G5P[7]** | **EU542703** | **99.93** | **99.93** |
|  | A2 | RVA/Human-tc/USA/DS-1/1976/G2P1B[4] | EF672578 | 65.94 | 65.94 |
|  | A8 | RVA/Horse-tc/GBR/H-1/1975/G5P[7] | JQ309141 | 70.86 | 70.86 |
|  | A8 | RVA/Pig-tc/MEX/YM/1983/G11P9[7] | D38154 | 70.20 | 70.20 |
|  | A8 | RVA/Pig-tc/USA/Gottfried/1983/G4P[6] | U08431 | 69.94 | 69.94 |
| NSP2 | **N1** | **RVA/Cow-tc/KOR/K5/2004/G5P[7]** | **JX971576** | **-** | **99.58** |
|  | **N1** | **RVA/Pig-tc/KOR/K71/2006/G5P[7]** | **JX971585** | **99.58** | **-** |
|  | **N1** | **RVA/Cow-tc/KOR/KJ246/2006/G8P[7]** | **FJ206155** | **99.89** | **99.68** |
|  | **N1** | **RVA/Cow-tc/KOR/KJ330-1/2006/G8P[7]** | **FJ206158** | **99.89** | **99.68** |
|  | **N1** | **RVA/Cow-tc/KOR/KJ338-1/2006/G8P[7]** | **FJ206160** | **99.89** | **99.68** |
|  | **N1** | **RVA/Cow-tc/KOR/K8/2005/G5P[7]** | **EU542709** | **99.89** | **99.68** |
|  | **N1** | **RVA/Human-tc/USA/Wa/1974/G1P1A[8]** | **L04534** | **86.42** | **86.18** |
|  | **N1** | **RVA/Pig-tc/USA/OSU/1977/G5P9[7]** | **X06722** | **99.58** | **88.37** |
|  | **N1** | **RVA/Horse-tc/GBR/H-1/1975/G5P[7]** | **JQ309142** | **86.44** | **86.20** |
|  | **N1** | **RVA/Panda-tc/CHN/CH-1/2008/G1P[7]** | **GU188281** | **99.79** | **99.58** |
|  | **N1** | **RVA/Cow-tc/KOR/KJ25-1/2006/G8P[7]** | **FJ206120** | **99.89** | **99.68** |
|  | **N1** | **RVA/Pig-tc/VEN/A253/1988/G11P9[7]** | **EF990696** | **94.43** | **94.20** |
|  | **N1** | **RVA/Pig-tc/VEN/A131/1988/G3P9[7]** | **EF990688** | **91.12** | **90.88** |
|  | **N1** | **RVA/Pig-tc/MEX/YM/1983/G11P9[7]** | **GU199517** | **92.85** | **92.62** |
|  | **N1** | **RVA/Pig-tc/USA/Gottfried/1983/G4P[6]** | **GU199489** | **97.11** | **96.90** |
|  | **N1** | **RVA/Pig-tc/KOR/PRG942/2006/G9P[23]** | **JF796719** | **92.87** | **93.08** |
|  | **N1** | **RVA/Pig-tc/KOR/PRG9121/2006/G9P[7]** | **JF796730** | **87.32** | **87.32** |
|  | N2 | RVA/Human-tc/USA/DS-1/1976/G2P1B[4] | EF672580 | 79.94 | 79.95 |
|  | N2 | RVA/Cow-tc/KOR/KJ9-1/2006/G6P[7] | FJ206108 | 81.83 | 81.57 |
| NSP3 | **T1** | **RVA/Cow-tc/KOR/K5/2004/G5P[7]** | **JX971577** | **-** | **99.94** |
|  | **T1** | **RVA/Pig-tc/KOR/K71/2006/G5P[7]** | **JX971586** | **99.94** | **-** |
|  | **T1** | **RVA/Human-tc/USA/Wa/1974/G1P1A[8]** | **X81434** | **93.33** | **93.26** |
|  | **T1** | **RVA/Pig-tc/USA/Gottfried/1983/G4P[6]** | **X81430** | **97.43** | **97.49** |
|  | **T1** | **RVA/Horse-tc/GBR/H-1/1975/G5P[7]** | **JQ309143** | **98.38** | **98.44** |
|  | **T1** | **RVA/Pig-tc/USA/OSU/1977/G5P9[7]** | **X81431** | **99.94** | **100.00** |
|  | **T1** | **RVA/Panda-tc/CHN/CH-1/2008/G1P[7]** | **GU329525** | **99.88** | **99.94** |
|  | **T1** | **RVA/Cow-tc/KOR/KJ9-1/2006/G6P[7]** | **FJ206226** | **99.71** | **99.76** |
|  | **T1** | **RVA/Cow-tc/KOR/KJ246/2006/G8P[7]** | **FJ206190** | **99.82** | **99.88** |
|  | **T1** | **RVA/Cow-tc/KOR/KJ330-1/2006/G8P[7]** | **FJ206192** | **99.94** | **100.00** |
|  | **T1** | **RVA/Cow-tc/KOR/K8/2005/G5P[7]** | **EU542715** | **99.94** | **100.00** |
|  | **T1** | **RVA/Cow-tc/KOR/KJ338-1/2006/G8P[7]** | **FJ206193** | **99.94** | **100.00** |
|  | **T1** | **RVA/Cow-tc/KOR/KJ25-1/2006/G8P[7]** | **FJ206169** | **99.41** | **99.47** |
|  | **T1** | **RVA/Pig-tc/VEN/A253/1988/G11P9[7]** | **EF990697** | **97.10** | **97.17** |
|  | **T1** | **RVA/Pig-tc/VEN/A131/1988/G3P9[7]** | **EF990689** | **95.91** | **95.98** |
|  | **T1** | **RVA/Pig-tc/MEX/YM/1983/G11P9[7]** | **GU199518** | **92.99** | **92.99** |
|  | **T1** | **RVA/Pig-tc/KOR/PRG942/2006/G9P[23]** | **JF796720** | **87.69** | **87.79** |
|  | **T1** | **RVA/Pig-tc/KOR/PRG9121/2006/G9P[7]** | **JF796731** | **87.58** | **87.69** |
|  | T2 | RVA/Human-tc/USA/DS-1/1976/G2P1B[4] | EF672579 | 81.64 | 81.73 |
| NSP4 | **E1** | **RVA/Cow-tc/KOR/K5/2004/G5P[7]** | **JX971578** | **-** | **100.00** |
|  | **E1** | **RVA/Pig-tc/KOR/K71/2006/G5P[7]** | **JX971587** | **100.00** | **-** |
|  | **E1** | **RVA/Human-tc/USA/Wa/1974/G1P1A[8]** | **AF093199** | **86.25** | **86.25** |
|  | **E1** | **RVA/Panda-tc/CHN/CH-1/2008/G1P[7]** | **GU188282** | **92.60** | **92.60** |
|  | **E1** | **RVA/Pig-tc/VEN/A131/1988/G3P9[7]** | **AF144798** | **83.80** | **83.80** |
|  | **E1** | **RVA/Pig-tc/USA/Gottfried/1983/G4P[6]** | **GU199490** | **92.59** | **92.59** |
|  | **E1** | **RVA/Cow-tc/KOR/K8/2005/G5P[7]** | **EU542721** | **99.42** | **99.42** |
|  | **E1** | **RVA/Pig-tc/USA/OSU/1977/G5P9[7]** | **D88831** | **92.60** | **92.60** |
|  | **E1** | **RVA/Horse-tc/GBR/H-1/1975/G5P[7]** | **AF144800** | **90.78** | **90.78** |
|  | **E1** | **RVA/Cow-tc/KOR/KV0407/2004/G5P[7]** | **EU873006** | **99.04** | **99.04** |
|  | **E1** | **RVA/Cow-tc/KOR/KJ25-1/2006/G8P[7]** | **FJ206109** | **100.00** | **100.00** |
|  | **E1** | **RVA/Cow-tc/KOR/KJ246/2006/G8P[7]** | **FJ206159** | **100.00** | **100.00** |
|  | **E1** | **RVA/Cow-tc/KOR/KJ330-1/2006/G8P[7]** | **FJ206162** | **100.00** | **100.00** |
|  | **E1** | **RVA/Cow-tc/KOR/KJ338-1/2006/G8P[7]** | **FJ206163** | **100.00** | **100.00** |
|  | **E1** | **RVA/Pig-tc/VEN/A253/1988/G11P9[7]** | **AF144797** | **87.31** | **87.31** |
|  | **E1** | **RVA/Pig-tc/MEX/YM/1983/G11P9[7]** | **X69485** | **88.64** | **88.64** |
|  | **E1** | **RVA/Pig-tc/KOR/PRG942/2006/G9P[23]** | **JF796721** |  |  |
|  | **E1** | **RVA/Pig-tc/KOR/PRG9121/2006/G9P[7]** | **JF796732** |  |  |
|  | E2 | RVA/Human-tc/USA/DS-1/1976/G2P1B[4] | EF672582 | 75.72 | 75.72 |
|  | E2 | RVA/Cow-tc/KOR/KJ9-1/2006/G6P[7] | FJ206101 | 73.04 | 73.04 |
| NSP5 | **H1** | **RVA/Cow-tc/KOR/K5/2004/G5P[7]** | **JX971579** | **-** | **100.00** |
|  | **H1** | **RVA/Pig-tc/KOR/K71/2006/G5P[7]** | **JX971588** | **100.00** | **-** |
|  | **H1** | **RVA/Human-tc/USA/Wa/1974/G1P1A[8]** | **AF306494** | **93.67** | **93.67** |
|  | **H1** | **RVA/Pig-tc/USA/Gottfried/1983/G4P[6]** | **GU199491** | **97.77** | **97.77** |
|  | **H1** | **RVA/Cow-tc/KOR/K8/2005/G5P[7]** | **EU542727** | **100.00** | **100.00** |
|  | **H1** | **RVA/Pig-tc/USA/OSU/1977/G5P9[7]** | **X15519** | **98.98** | **98.98** |
|  | **H1** | **RVA/Horse-tc/GBR/H-1/1975/G5P[7]** | **JQ309144** | **97.42** | **97.42** |
|  | **H1** | **RVA/Panda-tc/CHN/CH-1/2008/G1P[7]** | **GU329526** | **99.49** | **99.49** |
|  | **H1** | **RVA/Cow-tc/KOR/KJ9-1/2006/G6P[7]** | **FJ206045** | **99.49** | **99.49** |
|  | **H1** | **RVA/Cow-tc/KOR/KJ25-1/2006/G8P[7]** | **FJ206059** | **100.00** | **100.00** |
|  | **H1** | **RVA/Cow-tc/KOR/KJ246/2006/G8P[7]** | **FJ206097** | **100.00** | **100.00** |
|  | **H1** | **RVA/Cow-tc/KOR/KJ330-1/2006/G8P[7]** | **FJ206099** | **100.00** | **100.00** |
|  | **H1** | **RVA/Cow-tc/KOR/KJ338-1/2006/G8P[7]** | **FJ206100** | **100.00** | **100.00** |
|  | **H1** | **RVA/Pig-tc/VEN/A253/1988/G11P9[7]** | **EF990698** | **93.72** | **93.72** |
|  | **H1** | **RVA/Pig-tc/VEN/A131/1988/G3P9[7]** | **EF990690** | **91.61** | **91.61** |
|  | **H1** | **RVA/Pig-tc/MEX/YM/1983/G11P9[7]** | **X69486** | **97.77** | **97.77** |
|  | **H1** | **RVA/Pig-tc/KOR/PRG942/2006/G9P[23]** | **JF796722** | **97.14** | **97.14** |
|  | **H1** | **RVA/Pig-tc/KOR/PRG9121/2006/G9P[7]** | **JF796733** | **97.98** | **97.98** |
|  | H2 | RVA/Human-tc/USA/DS-1/1976/G2P1B[4] | EF672583 | 80.47 | 80.47 |

^a^ The cut-off values of nucleotide percentage identities of each gene segment is as follows: VP7: 80%, VP4: 80%, VP6: 85%, VP1: 83%, VP2: 84%,VP3: 81%, NSP1: 79%, NSP2: 85%, NSP3: 85%, NSP4: 85% and NSP5: 91%.

^b^ The strains having high nucleotide identities with the Korean strains, are written in bold.

^c^ Nucleotide sequences were not submitted in GenBank.
