# Supplementary material for: Different virulence of porcine and porcine-like bovine rotavirus strains with genetically nearly identical genomes in piglets and calves
Source: Vet Res. 2013 Oct 1;44(1):88. doi: 10.1186/1297-9716-44-88 (PMC3851489; doi:10.1186/1297-9716-44-88)
Supplement: Additional file 3 — Comparison of genotype constellation of porcine and porcine-like G5P[7] strains with other known reference genotypes. The proposed classification by the RCWG was applied to the structural and non-structural protein encoding genes for porcine K71 and bovine K5. Green was used for Wa-like strains, while red indicates DS-1 like strains. Blue and dark green were used for some typical porcine segments, while purple and pink were used for bovine-like segments. [file 1297-9716-44-88-S3.docx]

| **Virus/Species/Contry/Strain/**  **Year of isolation/Genotypes** | **Species** | **Genome constellation** | | | | | | | | | | |
| --- | --- | --- | --- | --- | --- | --- | --- | --- | --- | --- | --- | --- |
|  |  | **VP7** | **VP4** | **VP6** | **VP1** | **VP2** | **VP3** | **NSP1** | **NSP2** | **NSP3** | **NSP4** | **NSP5** |
| RVA/Human-tc/USA/Wa/1974/G1P1A[8] | Human | G1 | P[8] | I1 | R1 | C1 | M1 | A1 | N1 | T1 | E1 | H1 |
| RVA/Panda-tc/CHN/CH-1/2008/G1P[7] | Panda | G1 | P[7] | I5 | R1 | C1 | M1 | A1 | N1 | T1 | E1 | H1 |
| RVA/Human-tc/USA/DS-1/1976/G2P1B[4] | Human | G2 | P[4] | I2 | R2 | C2 | M2 | A2 | N2 | T2 | E2 | H2 |
| RVA/Pig-tc/VEN/A131/1988/G3P9[7] | Porcine | G3 | P[7] | I5 | R1 | C2 | M1 | A1 | N1 | T1 | E1 | H1 |
| RVA/Pig-tc/USA/Gottfried/1983/G4P[6] | Porcine | G4 | P[6] | I1 | R1 | C1 | M1 | A8 | N1 | T1 | E1 | H1 |
| **RVA/Cow-tc/KOR/K5/2004/G5P[7]** | **Bovine** | **G5** | **P[7]** | **I1** | **R1** | **C1** | **M1** | **A1** | **N1** | **T1** | **E1** | **H1** |
| **RVA/Pig-tc/KOR/K71/2006/G5P[7]** | **Porcine** | **G5** | **P[7]** | **I1** | **R1** | **C1** | **M1** | **A1** | **N1** | **T1** | **E1** | **H1** |
| RVA/Cow-tc/KOR/K8/2005/G5P[7] | Bovine | G5 | P[7] | *I1* | *R1* | *C1* | *M1* | A1 | N1 | T1 | E1 | H1 |
| RVA/Pig-tc/USA/OSU/1977/G5P9[7] | Porcine | G5 | P[7] | I5 | R1 | C1 | M1 | A1 | N1 | T1 | E1 | H1 |
| RVA/Horse-tc/GBR/H1/1975/G5P[7] | Equine | G5 | P[7] | I5 | R1 | C1 | M1 | A8 | N1 | T1 | E1 | H1 |
| RVA/Pig-xx/CHN/JL94/XXXX/G5P[7] | Porcine | G5 | P[7] | I5 |  |  |  |  |  |  |  |  |
| RVA/Cow-tc/KOR/KV0407/2004/G5P[7] | Bovine | G5 | P[7] | I5 |  |  |  |  |  |  | E1 |  |
| RVA/Pig-tc/KOR/85-1/2006/G5P[7] | Porcine | G5 | *P[7]* |  |  |  |  |  |  |  |  |  |
| RVA/Pig-tc/KOR/B-1/2006/G5P[7] | Porcine | G5 | *P[7]* |  |  |  |  |  |  |  |  |  |
| RVA/Pig-tc/KOR/150-1/2006/G5P[7] | Porcine | G5 | *P[7]* |  |  |  |  |  |  |  |  |  |
| RVA/Pig-tc/KOR/122-1/2006/G5P[7] | Porcine | G5 | *P[7]* |  |  |  |  |  |  |  |  |  |
| RVA/Cow-tc/KOR/KJ9-1/2006/G6P[7] | Bovine | G6 | P[7] | I2 | R2 | C1 | M2 | *A1* | N2 | T1 | E2 | H1 |
| RVA/Cow-tc/KOR/KJ25-1/2006/G8P[7] | Bovine | G8 | P[7] | I5 | R1 | C1 | M2 | *A1* | N1 | T1 | E1 | H1 |
| RVA/Cow-tc/KOR/KJ330-1/2006/G8P[7] | Bovine | G8 | *P[7]* | *I5* | *R1* | *C1* | *M2* | A1 | N1 | T1 | E1 | H1 |
| RVA/Cow-tc/KOR/KJ246/2006/G8P[7] | Bovine | G8 | *P[7]* | *I5* | *R1* | *C1* | *M2* | A1 | N1 | T1 | E1 | H1 |
| RVA/Cow-tc/KOR/KJ338/2006/G8P[7] | Bovine | G8 | *P[7]* | *I5* | *R1* | *C1* | *M2* | A1 | N1 | T1 | E1 | H1 |
| RVA/Pig-tc/VEN/A253/1988/G11P9[7] | Porcine | G11 | P[7] | I5 | R1 | C2 | M1 | A1 | N1 | T1 | E1 | H1 |
| RVA/Pig-tc/MEX/YM/1983/G11P9[7] | Porcine | G11 | P[7] | I5 | R1 | C1 | M1 | A8 | N1 | T1 | E1 | H1 |

**Additional file 3 Comparison of genotype constellation of porcine and porcine-like G5P[7] strains with other known reference genotypes.**
