# Supplementary figures and images for: Different virulence of porcine and porcine-like bovine rotavirus strains with genetically nearly identical genomes in piglets and calves
Source: Vet Res. 2013 Oct 1;44(1):88. doi: 10.1186/1297-9716-44-88 (PMC3851489; doi:10.1186/1297-9716-44-88)

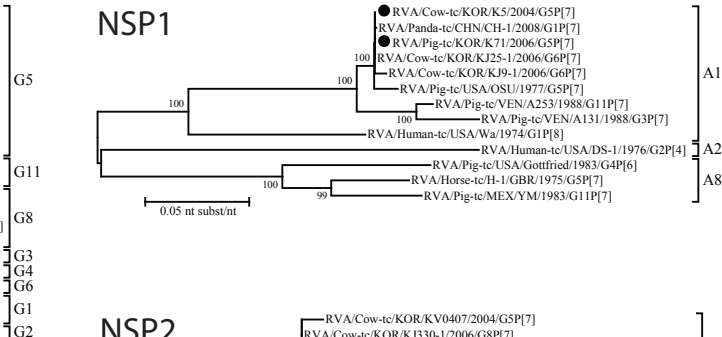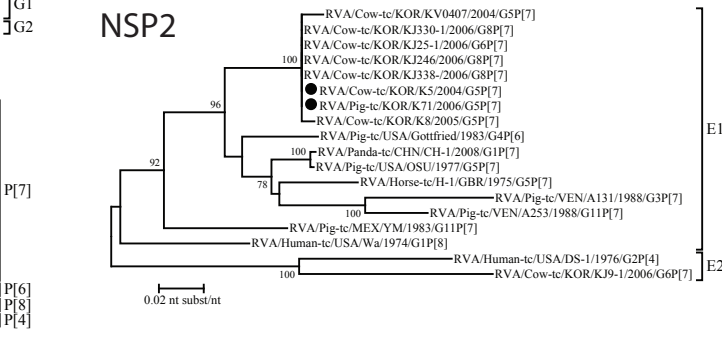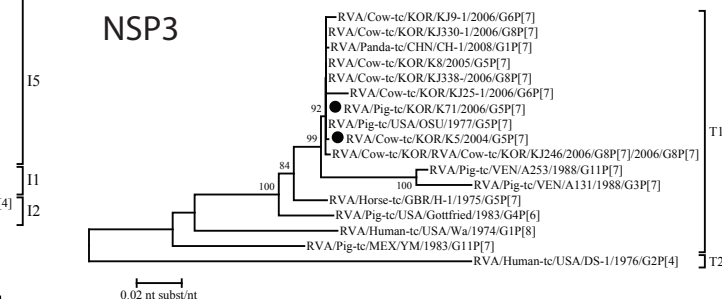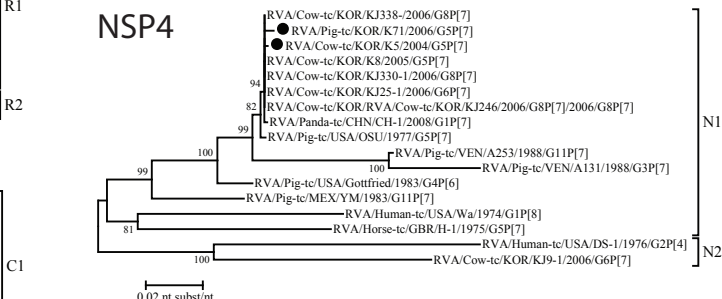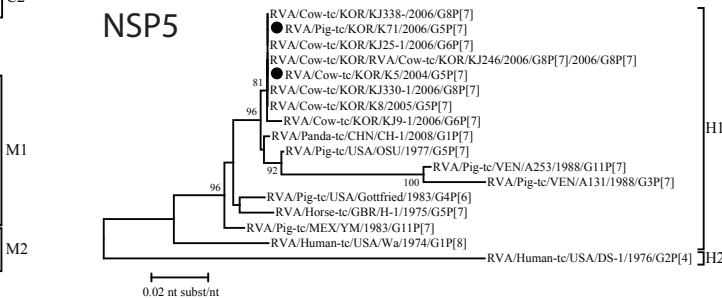

Supplement: Additional file 4 — Phylogenentic trees based on the full length nucleotide sequence of the 11 genomic segments of porcine G5P[7] K71 and porcine-like bovine G5P[7] K5 strains in comparison with reference RVA strains. The full length sequence of the 11 segments of porcine G5P[7] K71 and porcine-like bovine G5P[7] K5 strains were aligned, and phylogenetic trees were constructed using the neighbor-joining method with 1000 bootstrap replicates. Genetic distances were calculated using Kimura-2 correction parameter at the nucleotide level. [file 1297-9716-44-88-S4.pdf]
