# Supplementary material for: Different virulence of porcine and porcine-like bovine rotavirus strains with genetically nearly identical genomes in piglets and calves
Source: Vet Res. 2013 Oct 1;44(1):88. doi: 10.1186/1297-9716-44-88 (PMC3851489; doi:10.1186/1297-9716-44-88)
Supplement: Additional file 6 — Summary of the histopathological findings in the small intestine of the colostrum-deprived calves inoculated with a porcine G5P[7] K71 strain. The average villi/crypt (V/C) ratio plus the grade of epithelial cell desquamation of 10 randomly selected villi and crypt were used to determine the histopathological lesion in the small intestine of colostrum-deprived calves inoculated with porcine K71 strain. Indirect immunofluorescence assay with monoclonal antibody against the VP6 protein of strain OSU was conducted to measure the antigen distribution in the small intestine. [file 1297-9716-44-88-S6.docx]

**Additional file 6 Summary of the histopathological findings in the small intestine of the colostrums-deprived calves inoculated with a porcine G5P[7] K71 strain.**

| Calf  No. | Inoculum  (Days old) | dpi at euthanasia | Duodenum | |  | Jejunum | |  | Ileum | |
| --- | --- | --- | --- | --- | --- | --- | --- | --- | --- | --- |
|  |  |  | Lesion score^a^ | RVA Ag  distribution^b^ |  | Lesion score^a^ | RVA Ag  distribution^b^ |  | Lesion  score^a^ | RVA Ag  distribution^b^ |
| 1 | K71 (3) | 1 | 0.2 | 0.4 |  | 0.2 | 0.8 |  | 0 | 0.4 |
| 2 | K71 (3) | 3 | 0.1 | 0.2 |  | 0.2 | 0.4 |  | 0.1 | 0.4 |
| 3 | K71 (3) | 5 | 0.4 | 0 |  | 0.3 | 0 |  | 0.4 | 0 |
| 4 | K71 (3) | 7 | 0.2 | 0 |  | 0.2 | 0 |  | 0.3 | 0 |
| 5 | K71 (3) | 14 | 0.3 | 0 |  | 0.2 | 0 |  | 0.1 | 0 |
| 6 | Mock^a^ (3) | 2 | 0 | 0 |  | 0 | 0 |  | 0 | 0 |
| 7 | Inactivated  K71^b^ (3) | 3 | 0 | 0 |  | 0 | 0 |  | 0 | 0 |

^a^ The small intestinal changes were scored according to the average villi/crypt (V/C) ratio plus the grade of epithelial cell desquamation, which was measured as follows: V/C ratio, 0 = normal (V/C ≥ 6:1), 1 = mild (V/C = 5.0 to 5.9:1), 2 = moderate (V/C = 4.0 to 4.9:1), 3 = marked (V/C = 3.0 to 3.9:1), 4 = severe (V/C ≤ 3.0:1) and desquamation grade, 0 = normal (no desquamation), 1 = mild (cuboidal attenuation of tip villous epithelium), 2 = moderate (desquamation of upper villous epithelium), 3 = marked (desquamation of lower villous epithelium), 4 = severe (desquamation of crypt epithelium).

^b^ The antigen distribution in the small intestine was evaluated based on the number of antigen-positive cells in the villi, and was measured as follows: 0 = no positive cells, 1 = one to two positive cells in the villi, 2 = three to five positive cells scattered in the villi, 3 = many positive cells in the villi, 4 = positive reaction detected in almost all epithelial cells in the upper part of the villi.
