# Supplementary material for: Different virulence of porcine and porcine-like bovine rotavirus strains with genetically nearly identical genomes in piglets and calves
Source: Vet Res. 2013 Oct 1;44(1):88. doi: 10.1186/1297-9716-44-88 (PMC3851489; doi:10.1186/1297-9716-44-88)
Supplement: Additional file 7 — Summary of the histopathological findings in the small intestine of the colostrum-deprived piglets inoculated with a bovine G5P[7] K5 strain. The small intestinal changes were scored according to the average villi/crypt (V/C) ratio plus the grade of epithelial cell desquamation. The antigen distribution in the small intestine was evaluated based on the number of antigen-positive cells in the villi using an indirect immunofluorescence assay with monoclonal antibody against the VP6 protein of strain OSU. [file 1297-9716-44-88-S7.docx]

**Additional file 7 Summary of the histopathological findings in the small intestine of the colostrums-deprived piglets inoculated with a bovine G5P[7] K5 strain.**

| Piglet  No. | Inoculum  (Days old) | dpi at euthanasia | Duodenum | |  | Jejunum | |  | Ileum | |
| --- | --- | --- | --- | --- | --- | --- | --- | --- | --- | --- |
|  |  |  | Lesion score^a^ | RVA Ag  distribution^b^ |  | Lesion score^a^ | RVA Ag  distribution^b^ |  | Lesion  score^a^ | RVA Ag  distribution^b^ |
| 1 | K5 (3) | 1 | 1.0 | 3.0 |  | 0.9 | 2.8 |  | 0.8 | 3.0 |
| 2 | K5 (3) | 1 | 1.4 | 3.4 |  | 1.4 | 3.0 |  | 0.8 | 3.0 |
| 3 | K5 (3) | 3 | 3.2 | 2.6 |  | 2.9 | 2.4 |  | 2.8 | 2.4 |
| 4 | K5 (3) | 3 | 3.0 | 2.8 |  | 3.1 | 2.4 |  | 2.7 | 2.0 |
| 5 | K5 (3) | 5 | 3.4 | 1.6 |  | 3.0 | 1.4 |  | 3.0 | 1.4 |
| 6 | K5 (3) | 7 | 3.8 | 1.6 |  | 3.6 | 1.2 |  | 3.6 | 1.0 |
| 7 | K5 (3) | 14 | 3.7 | 0.6 |  | 3.6 | 0.4 |  | 3.2 | 0.4 |
| 8 | Mock^a^ (3) | 2 | 0 | 0 |  | 0 | 0 |  | 0 | 0 |
| 9 | Inactivated  K5^b^ (3) | 3 | 0 | 0 |  | 0 | 0 |  | 0 | 0 |

^a^ The small intestinal changes were scored according to the average villi/crypt (V/C) ratio plus the grade of epithelial cell desquamation, which was measured as follows: V/C ratio, 0 = normal (V/C ≥ 6:1), 1 = mild (V/C = 5.0 to 5.9:1), 2 = moderate (V/C = 4.0 to 4.9:1), 3 = marked (V/C = 3.0 to 3.9:1), 4 = severe (V/C ≤ 3.0:1) and desquamation grade, 0 = normal (no desquamation), 1 = mild (cuboidal attenuation of tip villous epithelium), 2 = moderate (desquamation of upper villous epithelium), 3 = marked (desquamation of lower villous epithelium), 4 = severe (desquamation of crypt epithelium).

^b^ The antigen distribution in the small intestine was evaluated based on the number of antigen-positive cells in the villi, and was measured as follows: 0 = no positive cells, 1 = one to two positive cells in the villi, 2 = three to five positive cells scattered in the villi, 3 = many positive cells in the villi, 4 = positive reaction detected in almost all epithelial cells in the upper part of the villi.
