# Supplementary material for: Different virulence of porcine and porcine-like bovine rotavirus strains with genetically nearly identical genomes in piglets and calves
Source: Vet Res. 2013 Oct 1;44(1):88. doi: 10.1186/1297-9716-44-88 (PMC3851489; doi:10.1186/1297-9716-44-88)
Supplement: Additional file 9 — Summary of the antigen distribution in the extraintestinal organs of the colostrum-deprived piglets inoculated with a porcine G5P[7] K71 strain. To determine the antigen distribution in the extraintestinal organs, the number of antigen-positive cells was evaluated using the indirect immunofluorescence assay with monoclonal antibody against the VP6 protein of strain OSU. Values represent the average that was calculated in 10 fields per section. [file 1297-9716-44-88-S9.docx]

**Additional file 9 Summary of the antigen distribution in the extraintestinal organs of the colostrums-deprived piglets inoculated with a porcine G5P[7] K71 strain.**

| Piglet  No. | Inoculum (Days old) | dpi at euthanasia | Distribution of RVA antigen in extraintestinal organs^a^ | | | |
| --- | --- | --- | --- | --- | --- | --- |
|  |  |  | Mesenteric  lymph node | Livers | Lungs | Choroid  plexus |
| 1 | K71 (3) | 1 | 2.8 | 1.0 | 0.4 | 0 |
| 2 | K71 (3) | 1 | 3.0 | 1.2 | 0.2 | 0 |
| 3 | K71 (3) | 3 | 3.6 | 2.6 | 0.6 | 0.4 |
| 4 | K71 (3) | 3 | 3.2 | 2.4 | 0.6 | 0.4 |
| 5 | K71 (3) | 5 | 2.2 | 1.8 | 0.4 | 0.2 |
| 6 | K71 (3) | 5 | 2.4 | 1.8 | 0.2 | 0.2 |
| 7 | K71 (3) | 7 | 2.0 | 1.2 | 0.2 | 0.2 |
| 8 | K71 (3) | 14 | 1.6 | 1.0 | 0 | 0 |
| 9 | Mock^a^ (3) | 2 | 0 | 0 | 0 | 0 |
| 10 | Inactivated  K71^b^ (3) | 3 | 0 | 0 | 0 | 0 |

^a^ The antigen distribution in the extraintestinal organs was evaluated based on the number of antigen-positive cells as follows: 0 = no positive cells, 1 = one to two positive cells, 2 = three to five positive cells scattered in tissue, 3 = many positive cells in tissues, 4 = positive in almost tissue.
