# Supplementary material for: Different virulence of porcine and porcine-like bovine rotavirus strains with genetically nearly identical genomes in piglets and calves
Source: Vet Res. 2013 Oct 1;44(1):88. doi: 10.1186/1297-9716-44-88 (PMC3851489; doi:10.1186/1297-9716-44-88)
Supplement: Additional file 10 — Summary of the antigen distribution in the extraintestinal organs of the colostrum-deprived calves inoculated with a porcine G5P[7] K71 strain. Major organs such as the lung, liver, mesenteric lymph node, and choroid plexus were subjected to indirect immunofluorescence assay with monoclonal antibody against the VP6 protein of strain OSU to determine if RVA antigen-positive cells were present. Ten fields per section were analyzed to calculate the average number of antigen-positive cells. [file 1297-9716-44-88-S10.docx]

**Additional file 10 Summary of the antigen distribution in the extraintestinal organs of the colostrums-deprived calves inoculated with a porcine G5P[7] K71 strain.**

| Calf  No. | Inoculum (Days old) | dpi at euthanasia | Distribution of RVA antigen in extraintestinal organs^a^ | | | |
| --- | --- | --- | --- | --- | --- | --- |
|  |  |  | Mesenteric  lymph node | Livers | Lungs | Choroid  plexus |
| 1 | K71 (3) | 1 | 0 | 0 | 0 | 0 |
| 2 | K71 (3) | 3 | 0 | 0 | 0 | 0 |
| 3 | K71 (3) | 5 | 0 | 0 | 0 | 0 |
| 4 | K71 (3) | 7 | 0 | 0 | 0 | 0 |
| 5 | K71 (3) | 14 | 0 | 0 | 0 | 0 |
| 6 | Mock^a^ (3) | 2 | 0 | 0 | 0 | 0 |
| 7 | Inactivated  K71^b^ (3) | 3 | 0 | 0 | 0 | 0 |

^a^ The antigen distribution in the extraintestinal organs was evaluated based on the number of antigen-positive cells as follows: 0 = no positive cells, 1 = one to two positive cells, 2 = three to five positive cells scattered in tissue, 3 = many positive cells in tissues, 4 = positive in almost tissue.
