# Supplementary material for: Different virulence of porcine and porcine-like bovine rotavirus strains with genetically nearly identical genomes in piglets and calves
Source: Vet Res. 2013 Oct 1;44(1):88. doi: 10.1186/1297-9716-44-88 (PMC3851489; doi:10.1186/1297-9716-44-88)
Supplement: Additional file 11 — Summary of the antigen distribution in the extraintestinal organs of the colostrum-deprived piglets inoculated with a bovine G5P[7] K5 strain. The antigen distribution in the extraintestinal organs was evaluated based on the number of antigen-positive cells. To calculate the number positive cells in the organs or tissues, 10 fields per section were analyzed with indirect immunofluorescence assay with monoclonal antibody against the VP6 protein of strain OSU. [file 1297-9716-44-88-S11.docx]

**Additional file 11 Summary of the antigen distribution in the extraintestinal organs of the colostrums-deprived piglets inoculated with a bovine G5P[7] K5 strain.**

| Piglet  No. | Inoculum (Days old) | dpi at euthanasia | Distribution of RVA antigen in extraintestinal organs^a^ | | | |
| --- | --- | --- | --- | --- | --- | --- |
|  |  |  | Mesenteric  lymph node | Livers | Lungs | Choroid  plexus |
| 1 | K5 (3) | 1 | 3.6 | 0.6 | 0 | 0 |
| 2 | K5 (3) | 1 | 3.8 | 0.4 | 0 | 0 |
| 3 | K5 (3) | 3 | 3.2 | 2.2 | 0.2 | 0.2 |
| 4 | K5 (3) | 3 | 3.0 | 2.0 | 0.4 | 0.6 |
| 5 | K5 (3) | 5 | 2.4 | 1.4 | 0.2 | 0.2 |
| 6 | K5 (3) | 7 | 2.0 | 1.0 | 0.2 | 0.2 |
| 7 | K5 (3) | 14 | 2.0 | 0.6 | 0 | 0 |
| 8 | Mock^a^ (3) | 2 | 0 | 0 | 0 | 0 |
| 9 | Inactivated  K5^b^ (3) | 3 | 0 | 0 | 0 | 0 |

^a^ The antigen distribution in the extraintestinal organs was evaluated based on the number of antigen-positive cells as follows: 0 = no positive cells, 1 = one to two positive cells, 2 = three to five positive cells scattered in tissue, 3 = many positive cells in tissues, 4 = positive in almost tissue.
