# Supplementary material for: Different virulence of porcine and porcine-like bovine rotavirus strains with genetically nearly identical genomes in piglets and calves
Source: Vet Res. 2013 Oct 1;44(1):88. doi: 10.1186/1297-9716-44-88 (PMC3851489; doi:10.1186/1297-9716-44-88)
Supplement: Additional file 12 — Summary of the antigen distribution in the extraintestinal organs of the colostrum-deprived calves after inoculation with a bovine G5P[7] K5 strain. The data represent the average value of antigen-positive cells in the mesenteric lymph nodes, livers, lungs, and choroid plexuses of calves inoculated with bovine K5 strain. Ten fields per section were analyzed to calculate the average number of antigen-positive cells with indirect immunofluorescence assay with monoclonal antibody against the VP6 protein of strain OSU. [file 1297-9716-44-88-S12.docx]

**Additional file 12 Summary of the antigen distribution in the extraintestinal organs of the colostrums-deprived calves after inoculation with a bovine G5P[7] K5 strain.**

| Calf  No. | Inoculum (Days old) | dpi at euthanasia | Distribution of RVA antigen in extraintestinal organs^a^ | | | |
| --- | --- | --- | --- | --- | --- | --- |
|  |  |  | Mesenteric  lymph node | Livers | Lungs | Choroid  plexus |
| 1 | K5 (3) | 1 | 3.0 | 1.2 | 0 | 0 |
| 2 | K5 (3) | 1 | 2.8 | 1.0 | 0 | 0 |
| 3 | K5 (3) | 3 | 3.6 | 2.4 | 0.6 | 0.2 |
| 4 | K5 (3) | 3 | 3.6 | 2.2 | 0.8 | 0.4 |
| 5 | K5 (3) | 5 | 2.8 | 1.8 | 0.4 | 0.2 |
| 6 | K5 (3) | 5 | 2.6 | 1.4 | 0.2 | 0.2 |
| 7 | K5 (3) | 7 | 2.2 | 1.0 | 0.2 | 0.0 |
| 8 | K5 (3) | 14 | 1.4 | 0.2 | 0 | 0 |
| 9 | Mock^a^ (3) | 2 | 0 | 0 | 0 | 0 |
| 10 | Inactivated  K5^b^ (3) | 3 | 0 | 0 | 0 | 0 |

^a^ The antigen distribution in the extraintestinal organs was evaluated based on the number of antigen-positive cells as follows: 0 = no positive cells, 1 = one to two positive cells, 2 = three to five positive cells scattered in tissue, 3 = many positive cells in tissues, 4 = positive in almost tissue.
